# Oxidized Dopamine Acrylamide Primer to Achieve Durable Resin–Dentin Bonding

**DOI:** 10.34133/research.0101

**Published:** 2023-04-03

**Authors:** Leping Wu, Hui Shao, Yang Tao, Jingya Wu, Xinhui Wang, Qiufeng Nian, Shunli Zheng, Chris Ying Cao, Yuancong Zhao, Zheng Zhou, Hai Ming Wong, Quan-Li Li

**Affiliations:** ^1^ Key Laboratory of Oral Diseases Research of Anhui Province, College and Hospital of Stomatology, Anhui Medical University, 81 Meishan Road, Hefei 230032, China.; ^2^ The Affiliated Jiangning Hospital with Nanjing Medical University, Nanjing, Jiangsu 211100, China.; ^3^ Key Laboratory of Advanced Technology for Materials of Education Ministry, School of Materials Science and Engineering, Southwest Jiaotong University, Chengdu 610031, China.; ^4^ School of Dentistry, University of Detroit Mercy, Detroit, MI 48208-2576, USA.; ^5^ Faculty of Dentistry, The University of Hong Kong, 34 Hospital Road, The Prince Philip Dental Hospital, Hong Kong 999077, China.

## Abstract

The durability of the resin–dentin bonding interface is a key issue in clinical esthetic dentistry. Inspired by the extraordinary bioadhesive properties of marine mussels in a wet environment, we designed and synthetized N-2-(3,4-dihydroxylphenyl) acrylamide (DAA) according to the functional domain of mussel adhesive proteins. DAA’s properties of collagen cross-linking, collagenase inhibition, inducing collagen mineralization in vitro, and as a novel prime monomer for clinical dentin adhesion use, its optimal parameters, and effect on the adhesive longevity and the bonding interface’s integrity and mineralization, were evaluated in vitro and in vivo. The results showed that oxide DAA can inhibit the activity of collagenase and cross collagen fibers to improve the anti-enzymatic hydrolysis of collagen fibers and induce intrafibrillar and interfibrillar collagen mineralization. As a primer used in the etch-rinse tooth adhesive system, oxide DAA can improve the durability and integrity of the bonding interface by anti-degradation and mineralization of the exposed collagen matrix. Oxidized DAA (OX-DAA) is a promising primer for improving dentin durability; using 5% OX-DAA ethanol solution and treating the etched dentin surface for 30 s is the optimal choice when used as a primer in the etch-rinse tooth adhesive system.

## Introduction

A characteristic of modern clinical dentistry is minimally invasive tooth defects restoration based on composite resin bonding. However, the bonding strength between resin adhesives and dentin decreases remarkably with time, particularly in restorations that are in place for an extended period of time, raising serious concerns regarding the aging of resin-based restorations [1]. Half of all resin composite direct restorations are estimated to fail within 10 years, and replacing them takes up 60% of the dentist’s practice time [2,3]. Resin–dentin bonding primarily relies on the infiltration and diffusion of the adhesive monomer into the demineralized dentin collagen matrix, followed by monomer polymerization and curing in the collagen matrix, forming a mechanical locking structure called a hybrid layer composed of adhesive resin wrapping dentin collagen fibers [1,4]. However, a layer of demineralized collagen matrix at the bottom of the hybrid layer is always exposed, owing to a mismatch between the depth of demineralization and resin penetration. These exposed collagen fibers are vulnerable to degradation by endogenous (matrix metalloproteinases [MMPs] and cysteinase) and exogenous proteases, resulting in the destruction of the bonding interface [4]. Therefore, inhibiting the degradation of collagen fibers and maintaining the integrity of the hybrid layer are key factors for improving the durability of dentin–resin binding [5]. Furthermore, owing to the permeability and hydrophilicity of the hybrid layer, hydrolysis of the bond resin ester occurs in the water environment, which reduces the mechanical properties inside the hybrid layer and eventually leads to degradation of the binding interface.

The strategy to improve the durability of dentin adhesion may be summarized as follows: reducing the water in the bonding interface, such as ethanol wet bonding [6]; use of proteolytic enzyme/collagen hydrolysis enzyme inhibitors, such as ethylenediaminetetraacetic acid (EDTA), chlorhexidine, quaternary ammonium salt compounds, and tetracycline and its analogs [7–11]; collagen cross-linking agents, such as formaldehyde, glutaraldehyde, carbodiimide, proanthocyanidin, epigallocatechin gallate, and riboflavin [11–16]; and remineralizing the exposed collagen fibers in the hybrid layers to restore the multistage structure and mechanical properties of demineralized dentin [17,18]. However, the durability of the resin–dentin interface remains a challenge. Therefore, constructing a molecule with cross-linked collagen and inhibiting collagenase activity, as well as promoting the remineralization of demineralized dentin, may be an ideal candidate strategy to realize the durability of the resin–dentin bonding interface.

Mussels secrete protein-based adhesives using a network of byssal threads to secure themselves against wet rocks, while sandcastle worms secrete proteinaceous glue to bond sand grains and/or stones to build a protective tubular shell [19]. These adhesive proteins are rich in L-3,4-dihydroxyphenylalanine (DOPA), which plays an important role in extraordinarily robust underwater adhesion [20]. Therefore, DOPA or its derivatives, such as dopamine, containing synthetic mimic molecules have inspired many researchers to develop biomimetic adhesives for bonding in wet or underwater conditions [21,22]. Our previous research has shown that mussel adhesive protein may inhibit MMP activity and facilitate dentin adhesion [23], and a DOPA derivative of N-2-(3,4-dihydroxylphenyl) acrylamide (DAA) was designed and synthesized (Fig. S1) to effectively bind collagen; improve cross-linking degree, thermal stability, resistance to enzymatic hydrolysis, and mechanical properties of collagen; and also induce collagen intra- and interfibrillar mineralization [24,25]. In addition, DAA has an amide (–CO–NH–) group, which differs from the ester group (–CO–O–R–) in traditional acrylate and methacrylate adhesive monomers. The amide group is more resistant to hydrolysis than the ester group [1,26,27]. Simultaneously, the terminal carbon–carbon double bond (CH_2_=CH–) structure in the molecule can be copolymerized with the adhesive monomer to form a chemical bond. Therefore, we speculated that DAA, as a novel adhesive monomer, may form a chemical-micro-interlocking interface between the bonding resin and the dentin collagen matrix to achieve long-term interface stability by chemically bonding of collagen molecules, cross-linking of collagen fibers, anti-enzyme hydrolysis, and remineralization of exposed collagen fibers. However, as a new dental adhesive for clinical use, the following questions must be answered. First, the protein-based adhesive containing DOPA had a stronger adhesion ability under marine conditions with a pH value of approximately 8.8. DAA is also easily oxidized; does the oxidized DAA improve its adhesive ability? Second, the operative time for clinical dental adhesives is very limited, and does DAA reach the requirement? Third, what is the optimal concentration for clinical dental adhesion? Fourth, does DAA influence polymerization of the adhesive monomer?

## Results

### Characterization of DAA and oxidated DAA (OX-DAA) modified collagen

**Fig. 1.**
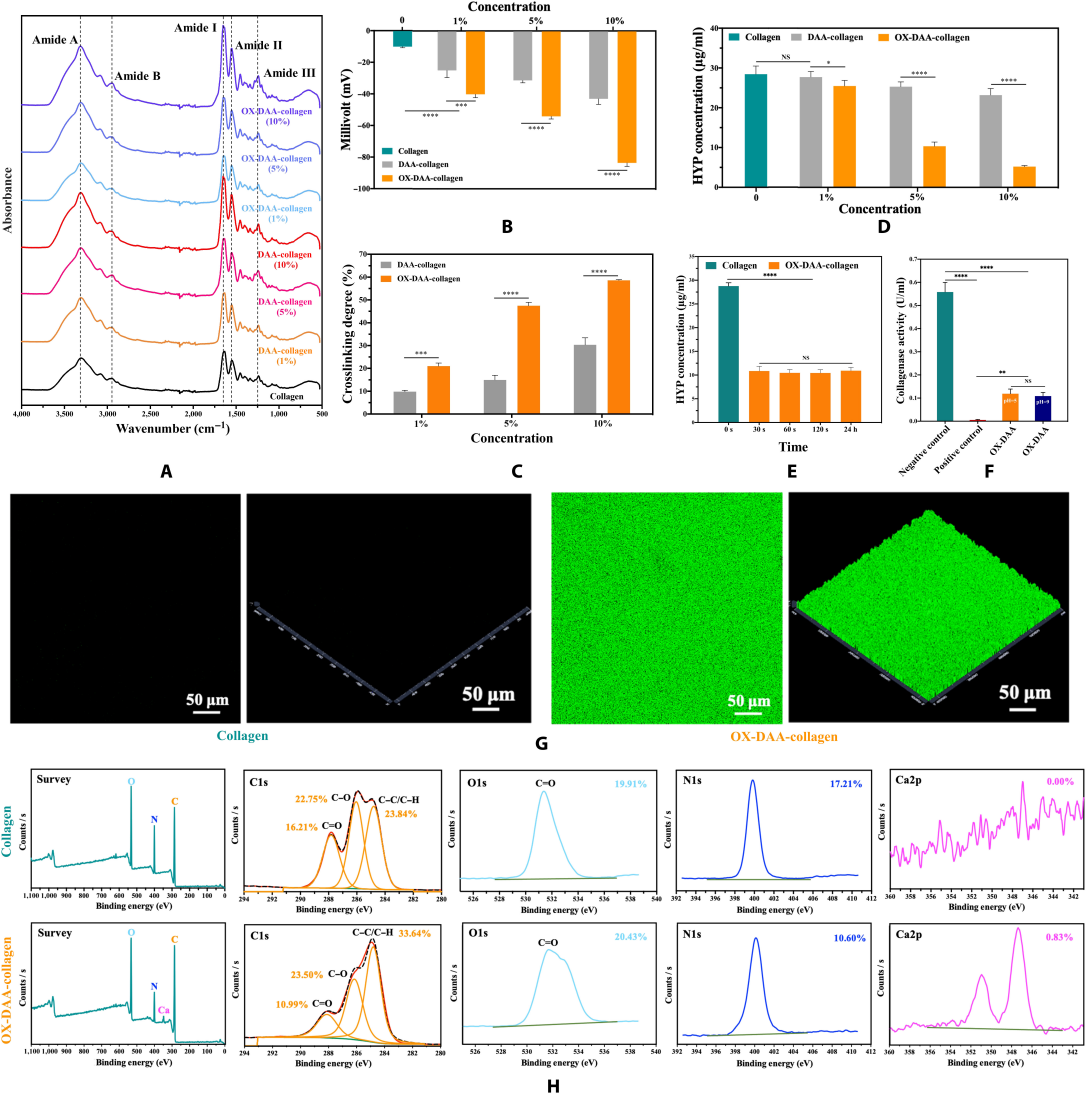
Characterization of collagen modified by DAA and OX-DAA. (A) ATR-FTIR spectra of untreated collagen membrane and collagen membranes treated with different concentrations of DAA and OX-DAA. (B) Zeta potential of untreated collagen membrane and collagen membranes treated with different concentrations of DAA and OX-DAA. (C) Cross-linking degree of collagen membrane treated with different concentrations of DAA and OX-DAA. (D) Enzymolysis solution’s HYP concentration of collagen membranes treated with different concentrations of DAA and OX-DAA. (E) Enzymolysis solution HYP concentration of collagen membrane treated with 5% OX-DAA different times. (F) Collagenase activity of positive control group, negative control group OX-DAA group (pH = 5), and OX-DAA group (pH = 9). (G) CLSM fluorescence images of collagen and OX-DAA-collagen membranes after immersed into the calcein-labeled CaCl_2_ solution. (H) XPS spectra of collagen and OX-DAA-collagen membranes after immersed into the CaCl_2_ solution. *P < 0.05, **P < 0.01, ***P < 0.001, ****P < 0.0001. NS, not significant.

The attenuated total reflection-Fourier transform infrared (ATR-FTIR) spectra of the interactions between DAA and oxidated DAA (OX-DAA) with collagen are shown in Fig. 1A. The characteristic peak parameters of the ATR-FTIR spectra of the collagen before and after treatment are shown in Table S1. Spectral analysis showed that the characteristic peak of the collagen triple-helix structure persisted after DAA and OX-DAA treatment. Due to cross-linking, the amide I band from 1,634 to 1,653 cm^−1^ and the amide II band from 1,548 to 1,552 cm^−1^ moved slightly, but the positions of the other collagen characteristic peaks did not change markedly. In addition, the absorption peak intensity of amide I of collagen was slightly increased after cross-linking with DAA and OX-DAA (each group’s collagen membrane had the same thickness).

The solid-surface zeta potentials of the untreated collagen, DAA-collagen, and OX-DAA-collagen membranes are shown in Fig. 1B. The zeta potential of the DAA-collagen membrane (ζ potential: 1%: −24.97 ± 4.52 mV, 5%: −31.33 ± 1.49 mV, and 10%: −43.00 ± 3.47 mV) and OX-DAA-collagen membrane (ζ potential: 1%: −40.09 ± 2.15 mV, 5%: −54.09 ± 1.65 mV, and 10%: −83.54 ± 2.26 mV) was markedly lower than untreated collagen membrane (ζ potential: −10.14 ± 0.67 mV), and with the increase of concentration of DAA and OX-DAA, the zeta potential value decreased more (*P* < 0.05). For the same concentration of DAA and OX-DAA, the zeta potential of the OX-DAA-collagen membrane was lower than that of the DAA-collagen membrane (*P* < 0.05).

The cross-linking degrees of the DAA-collagen and OX-DAA-collagen membranes are shown in Fig. 1C. The cross-linking degree increased in DAA-collagen membrane (1%: 9.86 ± 0.61%, 5%: 14.99 ± 2.06%, and 10%: 30.35 ± 3.05%) and OX-DAA-collagen membrane (1%: 21.01 ± 1.38%, 5%: 47.46 ± 1.49%, and 10%: 58.61 ± 0.29%) with the increase of concentration of DAA and OX-DAA (*P* < 0.05). For the same concentration of DAA and OX-DAA, the cross-linking degree of OX-DAA-collagen was greater than that of DAA-collagen (*P* < 0.05).

### Anti-enzymolysis ability of DAA-collagen and OX-DAA-collagen

Hydroxyproline (HYP) concentrations of DAA-collagen and OX-DAA-collagen membranes treated with different DAA concentrations (1%, 5%, and 10%) after enzymolysis are shown in Fig. 1D. The results showed that an increase in the concentrations of DAA and OX-DAA led to a decreased concentration of HYP, which corresponded to a better anti-enzymatic hydrolysis effect (*P* < 0.05). However, at the same concentration, OX-DAA treatment of collagen anti-enzymatic hydrolysis effect of OX-DAA was significantly better than that of the DAA treatment (*P* < 0.05). In addition, there was no difference between 1% DAA-treated collagen and untreated collagen in its anti-enzymatic hydrolysis effect (*P* > 0.05). Therefore, OX-DAA obviously improved the anti-enzymatic hydrolysis ability of collagen, whereas DAA showed only a weak contribution to anti-enzymatic hydrolysis. Although 10% OX-DAA showed better performance against enzymatic hydrolysis of collagen membrane, it was found in the experiment that when the OX-DAA concentration exceeded 5%, different DAA particles would appear in the solution. Therefore, 5% OX-DAA is the optimal concentration.

In order to facilitate the clinical application of DAA, the processing time of 5% OX-DAA interacting with collagen was optimized. Figure 1E shows the HYP concentration in each collagen membrane after OX-DAA treatment for different periods (30 s, 60 s, 120 s, and 24 h). Compared to the control group, the HYP concentration of the OX-DAA-collagen membrane decreased after enzymatic hydrolysis, but there was no difference between the different treatment time groups (*P* > 0.05). The experimental results showed that 5% OX-DAA treatment could obviously improve the anti-enzymatic performance of collagen membrane, and anti-enzymatic effect of collagen membrane was not affected by time after 30 s. Therefore, 30 s of OX-DAA treatment is the optimal clinical application time.

The effects of 5% OX-DAA on collagenase activity were also investigated. The collagenase activity of the positive control, negative control, and experimental groups is shown in Fig. 1F. The collagenase activity of the positive control group, OX-DAA group (pH = 5), and OX-DAA group (pH = 9) was significantly inhibited compared to that of the negative control group (*P* < 0.05). There was no difference in collagenase inhibitory activity between the OX-DAA group (pH = 5) and the OX-DAA group (pH = 9) (*P* > 0.05); however, it was lower than that of the positive control group (*P* < 0.05). The results showed that OX-DAA inhibited collagenase activity in both acidic and alkaline conditions.

### Calcium-binding ability of collagen and OX-DAA-collagen

DAA was oxidized to form a phenylquinone structure (Figs. S2 and S3) to give the OX-DAA-modified collagen a strong calcium ion binding capacity. We evaluated the calcium ion binding ability using confocal laser scanning microscopy (CLSM) and x-ray photoelectron spectroscopy (XPS). Collagen and OX-DAA-collagen membranes were immersed in a calcein-labeled CaCl_2_ solution for 2 h, then rinsed with deionized water, and thoroughly dried. Laser confocal microscopy observation of calcium fluorescence on the collagen membrane surface showed that no green fluorescence was observed on the surface of the bare collagen membrane, while the OX-DAA-collagen membrane showed intensive calcium fluorescence (Fig. 1F). XPS of collagen and OX-DAA-collagen membranes revealed that the unmodified collagen failed to detect the presence of calcium (the minimum detection limit of XPS was about 0.1 atom % [1,000 ppm]), while the atomic percentage of calcium in OX-DAA-collagen membranes was 0.83% (Fig. 1G). The above experimental results indicated that the unmodified collagen had almost no ability to bind calcium ions, whereas the OX-DAA-modified collagen had some ability to bind calcium ions, which may contribute to the mineralization of the collagen fibrils.

### Mineralization ability of collagen induced by OX-DAA

#### Characterization of collagen Intrafibrillar mineralization

**Fig. 2.**
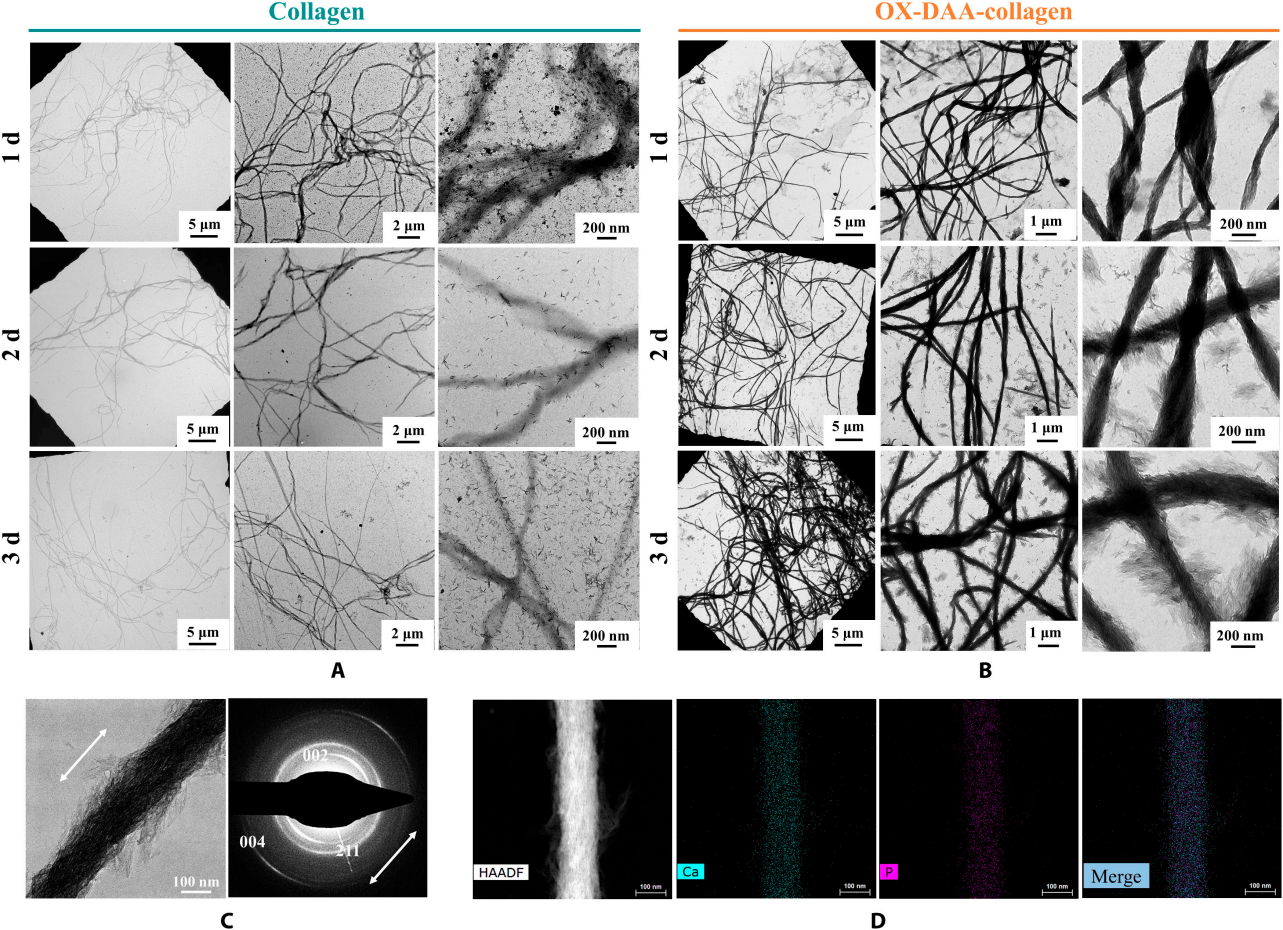
Evaluation of intrafibrillar mineralization. (A and B) TEM images of untreated collagen and OX-DAA-collagen mineralized for 1, 2, and 3 d. The images increase in magnification from left to right.(C) HRTEM image and SAED of OX-DAA-collagen after mineralizing for 2 d. (D) Dark-field image and elemental mapping of OX-DAA-collagen mineralization.

The effect of intrafibrillar mineralization was evaluated using transmission electron microscopy (TEM). Self-assembled collagen fibers showed the same 67-nm transverse line structure as native collagen (Fig. S4). The untreated collagen mineralized for 3 d is shown in Fig. 2A. After 24 h, untreated collagen fibers did not show intrafibrillar mineralization, and massive amorphous calcium phosphate (ACP) nanoparticles gathered around the surface of the collagen. After 48 h, ACP nanoparticles on the nickel-mesh carbon film and the collagen surface were transformed into hydroxyapatite (HA) crystals. After 72 h, the collagen fibers were still devoid of intrafibrillar mineralization, and a large number of HA crystals were present on and around the collagen surface. However, OX-DAA-collagen was almost completely mineralized after 24 h, and only a small amount of collagen fibers remained unmineralized (Fig. 2B). After 48 h, intrafibrillar mineralization was completed. The degree and diameter of the collagen fibers increased and mineralized HA crystals began to appear on the fiber surface (interfibrillar mineralization). After 72 h, the interfibrillar minerals of the collagen fibers were more abundant and coarser in diameter.

High-resolution TEM (HRTEM) showed that the *c*-axis of the mineral crystals of OX-DAA-collagen was aligned with the *c*-axis of collagen fibers (Fig. 2C). Selected area electron diffraction (SAED) patterns demonstrated that the collagen fiber mineral crystal was HA. The orientation of the HA (002) diffraction arches followed the coalignment of the crystallite *c*-axis with the long axis of the collagen fibrils (white double-headed arrows show the arrangement direction of the HA crystals). HRTEM element mapping showed within the collagen fibril and their relatively uniform structure by integration of the calcium phosphate mineral phase (Fig. 2D).

#### Characterization of collagen interfibrillar mineralization

**Fig. 3.**
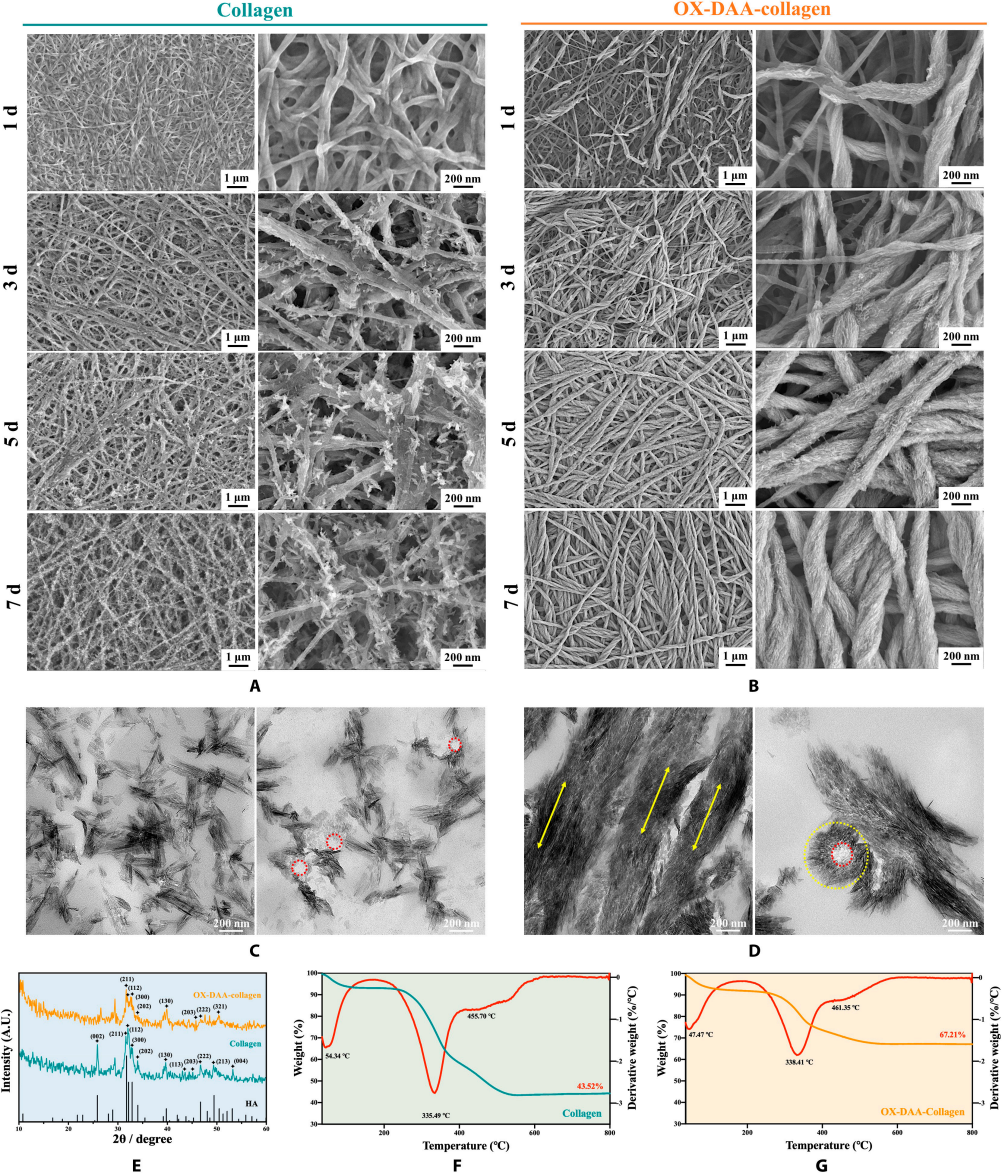
Evaluation of interfibrillar mineralization. (A and B) SEM images of untreated collagen and OX-DAA-collagen membrane mineralized for 1, 3, 5, and 7 d. The images increase in magnification from left to right. (C and D) TEM longitudinal section images of the untreated collagen and OX-DAA-collagen membranes mineralized for 7 d. (E) X-ray diffraction spectrum of untreated collagen and OX-DAA-collagen membrane (F and G) Thermodynamic analysis of untreated collagen and OX-DAA-collagen membrane mineralized for 7 d. A.U., arbitrary units.

The interfibrillar mineralization of collagen fibers begins at the end of intrafibrillar mineralization. The surface morphology of the interfibrillar mineralization of collagen fibers was observed using field emission scanning electron microscopy (FE-SEM). One day after mineralization, HA crystals did not appear on the surface of untreated collagen. Subsequently, with an increase in mineralization time, the HA crystals on the collagen surface increased, but the growth of HA crystals was disordered (Fig. 3A). In the OX-DAA group, collagen fibers were partially mineralized after 1 d of mineralization, and HA crystals grew along the collagen fibers (Fig. 3B). The *c*-axis orientation of HA followed the coalignment of collagen fibers along the long axis, and the morphology and structure of HA were the same as in biologically mineralized collagen fibers [28,29]. With the extension of mineralization time, the collagen fibers gradually became mineralized. On the third day, only a very small amount of collagen fibers was unmineralized, and on the fifth day, the collagen fibers were completely covered by interfibrillar HA crystals. On the seventh day, the diameter of mineralized collagen fibers increased substantially.

After 7 d of mineralization, TEM results showed a longitudinal section of the collagen membranes (Fig. 3C and D). The results showed that the interfibrillar HA crystals of untreated collagen were disorganized and oriented in all directions. At the same time, it could be seen from the longitudinal section that there was no intrafibrillar HA crystal in the untreated collagen fiber, as shown in Fig. 3C in the red broken circle. In the DAA-collagen group, the intrafibrillar and interfibrillar HA crystals *c*-axis orientation of HA followed the coalignment of collagen fibers along the long axis. When the direction of the collagen fibers’ long axis was parallel to the longitudinal sections, the HA crystals appeared as a line that was bundled together of intrafibrillar and interfibrillar (Fig. 3D; yellow double-headed arrows indicate the HA crystals arrangement direction) [25]. When the direction of the collagen fibers was perpendicular or at a certain angle to the longitudinal sections, the HA crystals appeared as dots that were bundled together by the intrafibrillar and interfibrillar (Fig. 3D [25]; red dotted circles show intrafibrillar HA crystals, and interfibrillar HA crystals are indicated between the red dotted circle and yellow dotted circle).

The minerals formed in collagen membranes were analyzed by x-ray diffraction (Fig. 3E). The diffraction peaks of the mineralized untreated collagen membrane and the OX-DAA-collagen membrane matched the HA standard peak. However, in the OX-DAA-collagen group, due to the interfibrillar *c*-axis orientation of HA following the coalignment of collagen fibers along the long axis, the sharp diffraction peaks (002) and (004) crystal planes disappeared [25].

The mineral content of the untreated collagen and OX-DAA-collagen membranes was measured by thermogravimetric analysis. After 7 d of mineralization, the results showed that mineral content of untreated collagen and OX-DAA-collagen membrane reached 43.52% and 67.21%, respectively (Fig. 3F and G). The degree of mineralization of the OX-DAA-collagen membrane was higher than that of the untreated collagen membrane. The thermodynamic degradation curve of the mineralized collagen membrane was divided into 3 stages: first, water evaporation inside the collagen fibers (~50 °C); second, thermodynamic degradation of collagen (~330 °C); and finally, oxidative decomposition of carbon into CO_2_ (~450 °C). The results showed that the thermodynamic degradation temperatures of the mineralized untreated collagen membrane and the OX-DAA-collagen membrane were basically the same, indicating that the mineral composition of the collagen membrane was similar.

#### OX-DAA induced demineralized dentin remineralization

**Fig. 4.**
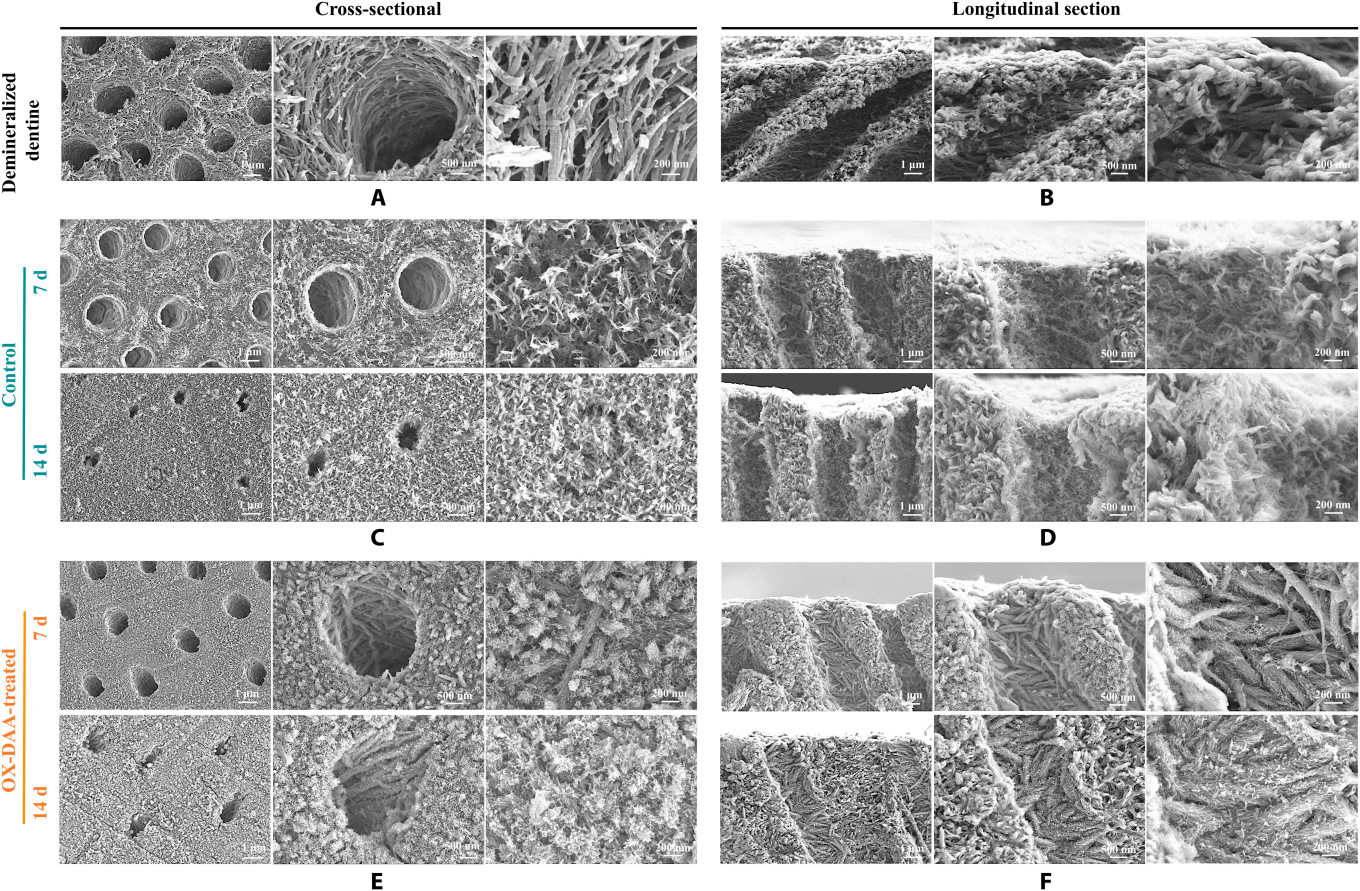
Evaluation of remineralization of demineralized dentin. (A and B) Demineralization dentin of cross-sectional and longitudinal section. (C and D) The cross-sectional and of longitudinal section of control group mineralized for 7 and 14 d. (E and F) The cross-sectional and of longitudinal section of OX-DAA-treated group mineralized for 7 and 14 d. The images increase in magnification from left to right.

After phosphoric acid etching, the dentinal tubules were opened and demineralized collagen fibers were exposed on the dentin surface and inner wall of the tubules. Depending on the mineral support, the demineralized collagen network slightly collapsed (Fig. 4A and B). After 7 d of remineralization, there was a lot of HA growth on the surface of both untreated demineralized dentin and demineralized collagen fibers within the dentinal tubules. However, the growth of these HA crystals was disordered (Fig. 4C and D). After 14 d of remineralization, the surface of the dentin was covered with a layer of HA crystals, the dentin tubules were partially occluded, and the openings became smaller (Fig. 4C and D). In the OX-DAA-treated group, after 7 d of remineralization, there was a large amount of HA growth on the demineralized collagen fibers (dentin surface and dentinal tubules). The *c*-axis orientation of HA followed the coalignment of collagen fibers along the long axis (Fig. 4E and F). After 14 d of remineralization, the collagen fibers were completely covered by HA crystals (Fig. 4E and F). At this time, HA gradually began to grow perpendicular to the demineralized dentin surface. The dentin tubules were partially blocked and their openings became smaller. The diameter of the mineralized collagen fibers inside the dentinal tubules increased, and the gaps between the fibers disappeared. The morphology of the OX-DAA-treated remineralized dentin was similar to that of natural dentin.

### DAA influence on adhesive polymerization and microtensile bond strength (μTBS) test

**Fig. 5.**
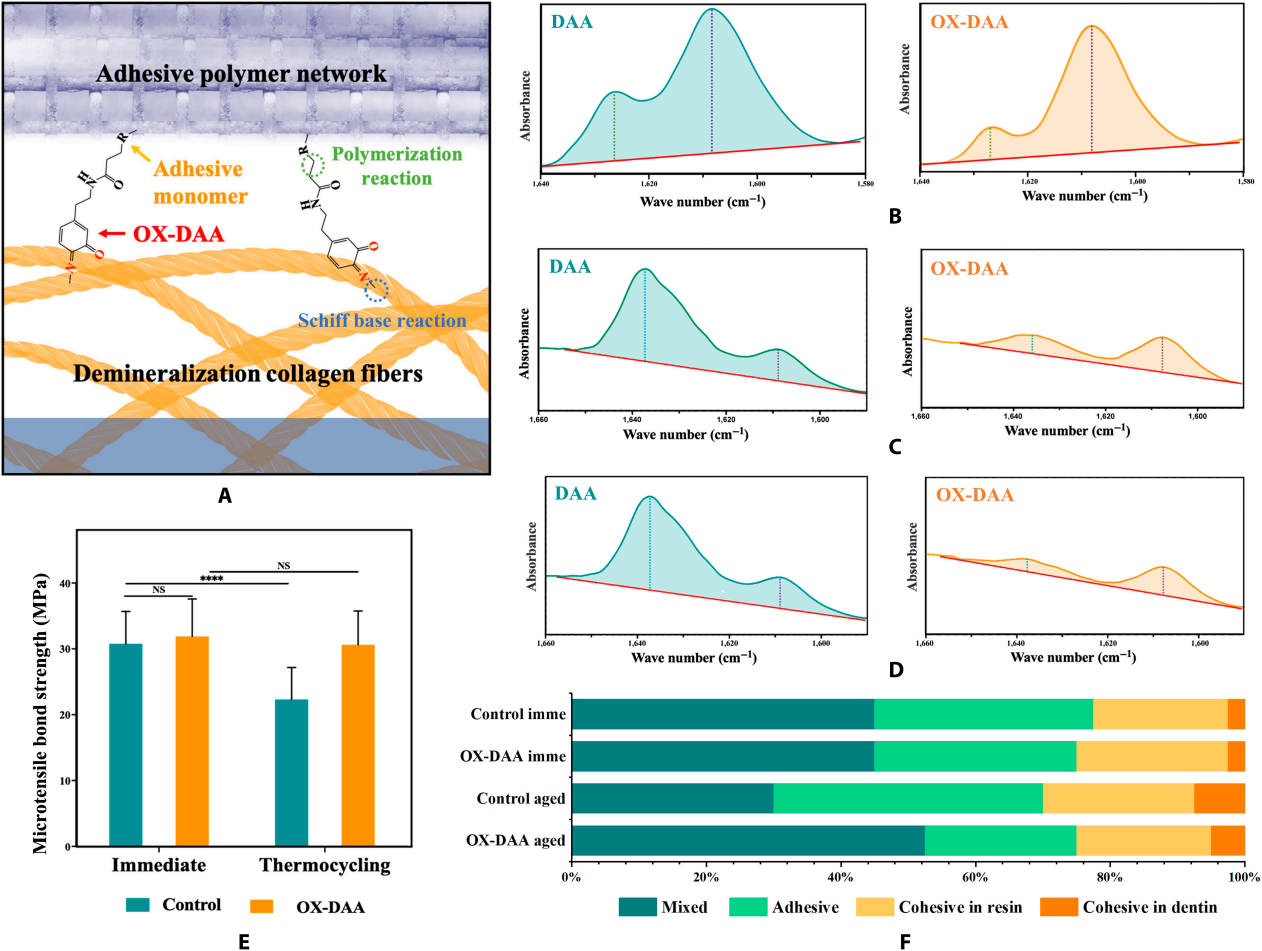
OX-DAA influence on adhesive polymerization and micro-tensile bond strength test. (A) Diagram of OX-DAA conjugation with collagen and adhesive together. (B) Representative spectrum of OX-DAA mixed camphorquinone before and after light-curing. (C) Representative spectrum of OX-DAA mixed commercial adhesive before and after light-curing. (D) Representative spectrum of commercial adhesive before and after light-curing. (E) Immediate and thermocycling microtensile bond strength of control and OX-DAA-treated groups. (F) Distribution of failure patterns (%) after μTBS test. The fracture types were classified as: adhesive (failure between the adhesive and dentin), cohesive in dentin (failure of the tooth substrate), cohesive in resin (failure of the composite resin), and mixed (adhesive and cohesive failure in resin). ****P < 0.0001, NS *P* > 0.05.

Before the microtensile bond strength (μTBS) test, we checked whether carbon–carbon double bonds (C=C) in the DAA molecule could be opened so that it could be polymerized with the C=C of the resin monomer. The polymerization of OX-DAA and the resin is shown in Fig. 5A. After 120 s of photocuring, OX-DAA molecules polymerized among each other, and the degree of conversion of OX-DAA molecule reached 48.76% (Fig. 5B), which was slightly higher than that of DAA molecule (40.60%; Figs. S4B and S5A). In addition, the effect of conversion of the resin was investigated. After 30 s of light curing, the degree of conversion of OX-DAA-mixed commercial resin reached 81.77% (Fig. 5C), slightly lower than that of commercial resin (84.62%; Fig. 5D), but higher than the degree of conversion of DAA-mixed commercial resin (57.60%; Fig. S4C and D).

The μTBS test results are shown in Fig. 5E. There was no difference in the resin–dentin bonding strength between the OX-DAA group (32.00 ± 5.73.92 MPa) and the control group (30.77 ± 4.92 MPa) during the immediate test (*P* > 0.05). After 5,000 cycles of thermocycling, the bonding strength of the control group (24.27 ± 6.71 MPa) decreased significantly (*P* < 0.05), while that of the DAA group (30.27 ± 5.84 MPa) did not change (*P* > 0.05). The experimental results showed that OX-DAA effectively improved the stability of the resin–dentin interface.

The fracture mode analysis of each group is shown in Fig. 5F. The fracture mode of each group is mainly mixed fracture, and there is no significant difference in the proportion of fracture modes among the groups (*P* > 0.05). However, in the control group, the proportion of adhesive fracture increased after aging. The proportion of mixed fracture in OX-DAA aging group is higher, while the proportion of adhesive fracture is lower.

### Evaluation of resin–dentin bonding interface quality

#### Morphological of bonding interfaces

**Fig. 6.**
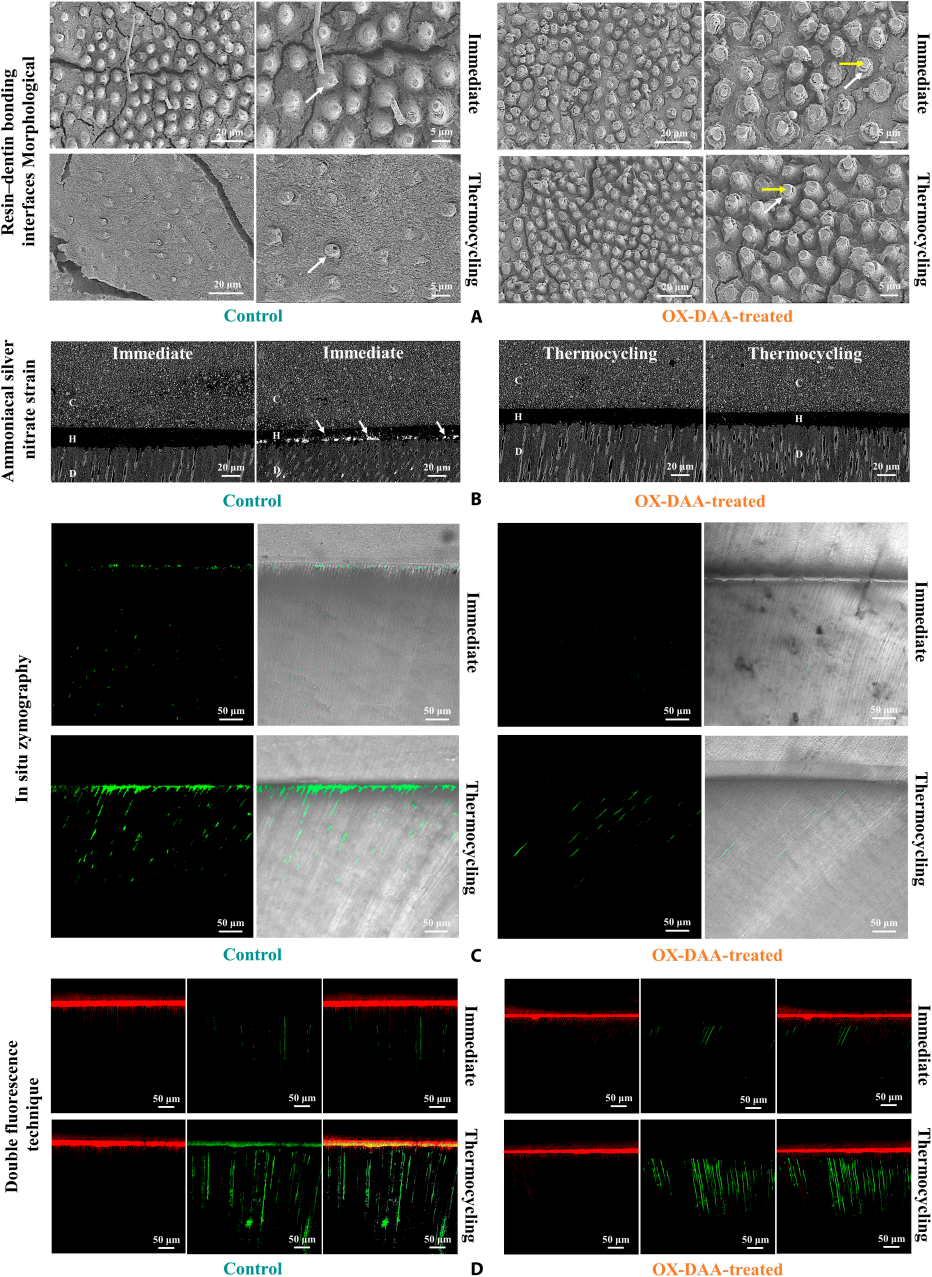
The evaluation influence of OX-DAA on the durability of resin–dentin bonding interface. (A) FE-SEM images of the resin–dentin bonding interfaces morphological. Yellow arrows, resin tags; white arrows, remaining hybrid layer. (B) Backscattered SEM images of the silver staining. White arrows, silver penetration; D, Dentin; H, adhesive layer; C, composite resin. (C) CLSM images of the in situ zymography. (D) CLSM images of the double fluorescence.

All dentin tissues (mineralized dentin and demineralized collagen matrix) were removed using phosphoric acid and hypochlorous acid, and only the polymerized resin was retained at the bonding interface (including adhesive resin infiltration into the hybrid layer formed by the collagen matrix and resin convex into the dentin tubules). FE-SEM revealed different morphological characteristics of the infiltrating resin at the bonding interface among the 4 groups (Fig. 6A). During immediate bonding, most of the resin tags in the control and OX-DAA groups were preserved, and the hybrid layer around the resin tags in the OX-DAA group was more intact than that in the control group. After thermocycling, most of the resin tags in the control group were destroyed, and a small number of resin tags of poor quality were preserved. However, the resin tags in the OX-DAA group remained intact, and there was no difference from those before thermocycling.

#### Ammoniacal silver nitrate stain of resin–dentin bonding interface

In the FE-SEM backscattering mode, the aggregation of silver particles at the nanoleakage on the adhesive surface is shown in Fig. 6B. There was no aggregation of silver particles at the bonding interface in the immediate bonding group, regardless of whether it was the control or OX-DAA group. After thermocycling, the entire bonding interface of the control group permeated with the agglomerated particles. However, there were no silver particles deposited at the bonding interface of the OX-DAA group, which is not immediately different from the OX-DAA group. The experimental results showed that the interconnectivity of nanocrystalline leakage in the control group increased after thermocycling. The experimental group effectively inhibited the expansion of nanoleakage.

#### In situ zymography of resin–dentin bonding interface

In situ zymography of the control and OX-DAA-treated groups of CLSM images are shown in Fig. 6C), with green fluorescence indicating endogenous MMPs active at these sites. In the immediate group, a small amount of green fluorescence was detected at the resin–dentin bonding interface and dentin tubules in the control group; almost no green fluorescence was observed at the bonding interface and dentin tubules in the OX-DAA group. After thermocycling, strong green fluorescence was detected at the interface and dentin tubules of the control group, indicating strong activity of endogenous MMPs at these sites. In the OX-DAA group, there was no green fluorescence at the bonding interface, but there was strong green fluorescence in the dentine tubules approximately 20 μm deep below the interface.

#### Double fluorescence of resin–dentin bonding interface

The double fluorescence CLSM images of the control group and the OX-DAA-treated group of CLSM images are shown in Fig. 6D. Rhodamine mixed into the adhesive showed red fluorescence at the hybrid layer of the adhesive interface and at the top of the dentin tubule, and fluorescein permeating into the pulp chamber showed green fluorescence. Rhodamine permeates dentin tubules from the bonding interface, whereas fluorescein diffuses in the opposite direction. The micro permeability of the resin–dentin interface was evaluated by combining double fluorescence images. In the immediate bonding, control, and OX-DAA groups, there was no yellow fluorescence at the dentin–resin bonding interface, indicating that no microleakage occurred. After thermocycling, the rhodamine-labeled hybrid layer in the control group was completely permeated by fluorescein sodium and showed bright yellow fluorescence, indicating microleakage at the interface. No yellow fluorescence was observed at the bonding interface of the OX-DAA group, that is, without nanoleakage occurred.

### Biocompatibility

**Fig. 7.**
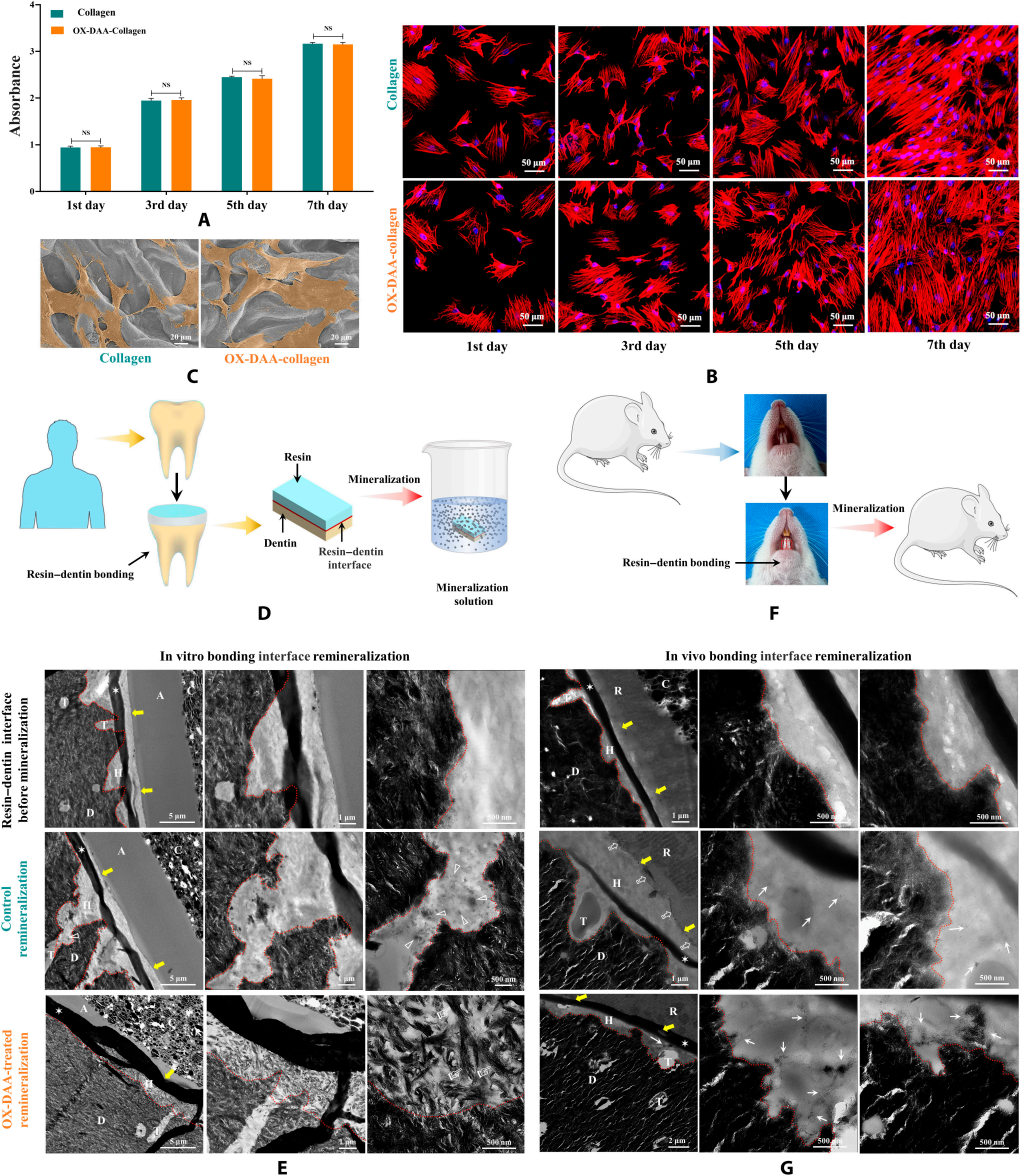
Cytocompatibility and resin–dentin interface remineralization. (A) The cell viability of Sprague Dawley bone marrow stem cells was measured by CCK-8 for 1, 3, 5, and 7 d. (B) Cell morphology stained with DAPI and phalloidin. Fluorescence images: cells in the collagen group and the OX-DAA-collagen group on the first, third, fifth, and seventh days. (C) SEM images of cells on the surface of the collagen and OX-DAA-collagen membranes on 7 d. NS *P* > 0.05; C, composite; A, adhesive; H, hybrid layer (between the dashed red line and the yellow arrow); D, mineralized intertubular dentin. T, dentinal tubule. (D) Schematic diagram of in vitro bonding interface remineralization. (E) TEM images of in vitro bonding interface remineralization for weeks. The images increase in magnification from left to right. (F) Schematic diagram of in vivo bonding interface remineralization. (G) TEM images of in vivo (SD rat) bonding interface remineralization for 12 d. The right and middle images are the local amplification of left image, respectively. The dark bands marked by asterisk were formed by fold accumulation during the preparation of ultrathin sections.

Cell viability was evaluated using a CCK-8 assay after 1, 3, 5, and 7 d (Fig. 7A). Cell viability was significantly increased from the first to the seventh day in both the untreated collagen and OX-DAA-collagen groups (*P* < 0.05), but there was no significant difference in cell viability between the 2 groups (*P* > 0.05). The TRITC-phalloidin images showed cellular pseudopods with active stretching and firm adhesion on the substrate (Fig. 7B). After 7 d of culture, the SEM images also showed that the cells presented numerous extending filopodia and lamellipodia formation and adhered well to the surface of the untreated collagen and OX-DAA-collagen (Fig. 7C). These results indicate that the OX-DAA molecules in OX-DAA-collagen were not toxic to Sprague Dawley bone marrow stem cells, thus having good biocompatibility.

### Bonding interface remineralization induced by OX-DAA

The remineralization of the resin–dentin bonding interface was evaluated by TEM in vitro and in vivo (Fig. 7D to G). The remineralization of the resin–dentin bonding interface in the in vitro model is shown in Fig. 7D. Before the resin–dentin bonding interface remineralization in vitro, TEM revealed a hybrid layer of completely demineralized dentin with an exposed collagen matrix (Fig. 7E). In the control group, remineralization could be vaguely discerned from the hybrid layers at 4 weeks (open arrowheads, Fig. 7E and Fig. S6A). A moderately high-magnification view shows regional mineral deposits (open arrowheads) of the resin-infiltrated collagen matrix. After 4 weeks, the hybrid layers were almost completely remineralized in the OX-DAA-treated group (Fig. 7E). Intrafibrillar remineralization was observed, with an orderly arrangement of nanoplatelets that revealed a pleaded, rope-like subfibrillar architecture of the collagen fibrils (pointer, Fig. 7E and Fig. S6B). There was minimal remineralization of the interfibrillar spaces, despite the presence of heavy remineralization within the collagen fibrils.

The remineralization of the resin–dentin bonding interface in the SD rat teeth model is shown in Fig. 7F). Immediately after resin binding, TEM revealed a hybrid layer of completely demineralized dentin devoid of intrafibrillar and interfibrillar mineral crystallites (Fig. 7G). At low magnification, the electron-dense distribution along the interface at the top of the hybrid layers was observed at 12 d (open arrow, Fig. 7G). A moderately high-magnification view showing a small number of dotted electron-dense in a hybrid layer, which may be the initial interfibrillar remineralization (arrows, Fig. S6C). After 12 d, the characteristic electron-dense mineral phase can be seen within the hybrid layer in the OX-DAA-treated group (arrows, Fig. 7G). These electron-dense mineral phases appeared as chains or clusters, which seemed to be aligned and oriented along the demineralized collagen fibers (arrows, Fig. S6D).

## Discussion

### DAA interaction with collagen and its parameter optimization for clinical application

Mussels and sandcastle worms have developed protein-based adhesives to adhere to various underwater substrates. Although the mechanisms are different and the exact reaction pathways are still under investigation, the adhesive proteins are all rich in DOPA, which is considered to be an important feature for underwater adhesion [19,30,31]. Given the seawater conditions, that is, pH 8 and high ionic strength, DOPA is readily oxidized to DOPA-quinone. DOPA can form hydrogen bonds with the substrate surface, whereas DOPA-quinone cannot [32,33]. The mussel foot deposits adhesive proteins onto target surfaces, creating an insulated reaction chamber to maintain location-specific redox control, such as low pH, low ionic strength, and high reducing poise, in which DOPA-quinone may be subsequently converted to *α*, *β*-dehydro-DOPA through tautomerization [34]. Both DOPA and *α*, *β*-dehydro-DOPA can interact with the adsorbed surface through a variety of mechanisms depending on the surface chemistry, such as bidentate H-bonds and hydrophobic, electrostatic, and π-cation interactions, and coordinate with metal ions and metal oxides to build up the adhesion [30–34]. Redox activity is driven by the difference between the high pH and O_2_ concentration of seawater, and the low pH and abundance of electron donors in the plaque. As time passes, the pH and O_2_ concentration increase, the adhesive proteins form complex coacervation by fluid–fluid phase separation, and DOPA is oxidized to DOPA-quinone, in which reactive o-quinones can undergo subsequent secondary reactions via nucleophilic addition or imine formation, which is essential for protein cross-linking and cohesion. Finally, the adhesive plaque microstructure forms and solidifies [30–34].

**Fig. 8.**
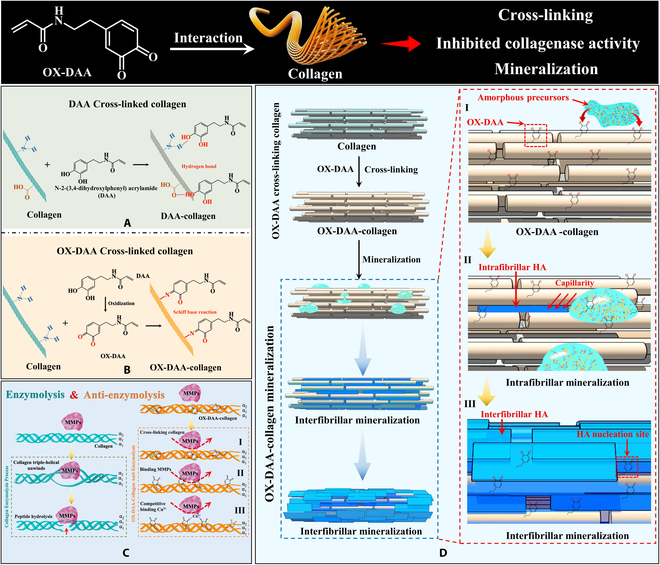
Schematic diagram of the mechanism of DAA-collagen by cross-linking, inhibiting collagenase activity, and inducing collagen fiber mineralization. (A) The mechanism of DAA and OX-DAA interaction with collagen. DAA cross-links collagen by hydrogen bonding. OX-DAA is covalently cross-linked with collagen side chain amino group by quinone group. (B) Collagen of enzymolysis and anti-enzymolysis mechanism. Steps involved in collagenolysis by MMPs: MMPs binds to triple-helical collagen and unwinds collagen. Unwound α-chain is then presented to the active site of the enzyme for peptide hydrolysis. Three mechanisms of OX-DAA-collagen anti-enzymolysis: OX-DAA cross-linked collagen to improve collagen stability; OX-DAA binding MMPs inhibited the activity of MMPs. OX-DAA and MMPs competitively bind Ca^2+^ and inhibit MMP activity. (C) The process of intrafibrillar and interfibrillar mineralization of collagen fibers induced by OX-DAA. (D) (I) OX-DAA attracts amorphous precursors to the collagen surface. (II) The amorphous precursors on collagen surface enters intrafibrillar by capillary force, forming intrafibrillar mineralization. (III) After the intrafibrillar mineralization is complete, the OX-DAA on collagen surface continues to attract Ca^2+^, further inducing the interfibrillar mineralization.

For both natural adhesive proteins and synthetic catechol-containing materials, catechol oxidation is detrimental to their adhesive ability, since the formed o-quinones are nonadhesive. In the present study, a catechol-containing adhesive, the DAA monomer, was used for dentin adhesion. Here, we used oxidized DAA, which acted as DOPA-quinone for protein cross-linking at the stage of adhesive plaque cohesion and solidification, to act with the demineralized collagen matrix to modify its properties and form a chemical bond. A novel functional adhesive monomer was used as a primer to modify the resin–dentin adhesion interface to improve the durability of resin–dentin bonding. DAA contains catechin and imine groups, which are similar to the dopamine and lysine functional groups in mussel adhesion protein molecules that interact with the collagen matrix. The highly active phenolic hydroxyl group in the DAA molecule has different reactivity with collagen under different conditions. Under acidic conditions, phenolic hydroxyl groups remain unoxidized, cross-linking DAA to collagen via amino acids containing –COOH and –NH_2_ side chains, and –OH interacts with –COOH and –NH_2_ mainly through hydrogen bonds (Fig. 8A) [24]. Under alkaline conditions, the phenolic hydroxyl group is easily oxidized to quinone, method of cross-linking of OX-DAA to collagen fibrils via amino acids containing –NH_2_ side chains, and –C=O interacts with –NH_2_ mainly through Schiff base reaction (Fig. 8B) [24].

The cross-linking mode between DAA and collagen under different conditions (nonoxidized/oxidized) has a significant effect on the performance of collagen. The results showed that the cross-linking of hydrogen bonds (a weak intermolecular interaction) between DAA and collagen had a much lower anti-enzymatic effect on collagen than covalent cross-linking of OX-DAA. As shown by our cross-linking degree study, OX-DAA markedly improved the cross-linking ability of collagen, whereas unoxidized DAA had a weak cross-linking ability of collagen (Fig. 1C). The binding site of collagenase and collagen is inside the collagen triple helix [35,36]. Strong and effective cross-linking makes the collagen triple-helix more stable [37], thus preventing collagenase from binding to the enzymatic hydrolysis site and effectively submitting the anti-enzymatic hydrolysis ability of collagen (Fig. 8CI). In addition to improving collagen cross-linking, inhibiting collagenase activity is the most direct way to improve the enzymatic hydrolytic ability of collagen (Fig. 8CII). The results showed that OX-DAA directly inhibited the collagenase activity. In addition, OX-DAA effectively inhibited collagenase activity in different pH (acidic/alkaline) environments (Fig. 1F). MMPs are endopeptidases that are synthesized and secreted as nonactive zymogens [38]. MMPs are activated by Zn^2+^ and Ca^2+^, and their activity is affected by pH [36]. In particular, its activity is stronger in acidic environments. However, the quinone group of OX-DAA has strong Ca^2+^ coordination ability and can competitively bind Ca^2+^ with MMPs, thereby inhibiting their activity (Fig. 8CIII). Therefore, OX-DAA can improve the anti-enzymatic ability of collagen by cross-linking collagen and inhibiting collagenase activity. OX-DAA is a more suitable primer for dentin bonding.

OX-DAA, as a primer before resin–dentin bonding, should be applied in accordance with the principles of clinical operation. First, the OX-DAA solution is more stable than DAA; therefore, OX-DAA should be easy to store. The higher the concentration of OX-DAA, the better the anti-enzymatic effect of collagen. However, intermolecular agglomerations of OX-DAA occurred, and the higher the concentration, the more unstable it became. It was found that the maximum stable concentration of OX-DAA was 5%, and OX-DAA solution with a concentration of more than 5% would have different degrees of precipitation after 24 h at room temperature. Therefore, 5% OX-DAA is the optimal concentration for clinical application. Second, the treatment time for collagen with OX-DAA is suitable. The clinical operation time is limited; the shorter the time, the more beneficial the clinical application. The anti-enzymatic hydrolysis ability of collagen membranes treated with 5% OX-DAA solution for 30 s, 60 s, 120 s, and 24 h was evaluated. The experimental results demonstrated no difference in the anti-enzymatic hydrolysis effect of collagen treated with OX-DAA for 30 s, 60 s, 120 s, and 24 h (Fig. 1E). Therefore, OX-DAA and collagen react rapidly and can fully react with collagen fibers within 30 s.

Therefore, 5% OX-DAA solution and treating the demineralized dentin collagen matrix for 30 s may be the optimal choice.

### OX-DAA improves the durability of resin–dentin bonding interface

As an adhesive primer monomer, DAA must be polymerized by itself and must not affect the polymerization of other adhesive monomers. It is considered that DAA-containing phenolic hydroxyl groups may inhibit monomer polymerization. The C=C conversion degree between the DAA molecules and the mixture of DAA with commercial adhesives was measured using a light initiator of camphor quinone. The results showed that the C=C bond in DAA could be broken and polymerized. Owing to the presence of phenolic hydroxyl groups, the C=C conversion rate of DAA was obviously lower than that of the commercial adhesive. However, the OX-DAA molecule is a quinone group; therefore, the inhibition of polymerization should be very weak. After OX-DAA was added to the commercial adhesive, the conversion degree of C=C was very close to that of the commercial adhesive, suggesting that OX-DAA had little effect on the polymerization of the commercial adhesive (Fig. 5C and D). Therefore, in the μTBS test, there was no difference in the immediate bonding tensile strength between the control and OX-DAA groups. However, the bonding strength of the control group decreased markedly after thermocycling, whereas the bonding strength of the OX-DAA group was not affected (Fig. 5E). OX-DAA exhibited excellent performance in terms of improving the durability of the bonding interface. In the fracture mode analysis experiment, mixed failure was dominant in both the experimental group and the control group, but the proportion of mixed failure was higher in the OX-DAA aging group, and the proportion of interface failure was lower (Fig. 5F). This result may be related to the formation of high-quality mixed layer promoted by OX-DAA.

The reason for this superior performance may be the protection of the integrity of the dentin–resin bonding interface. In the observation experiment of the dentin bonding interface morphology, the bonding interface of the OX-DAA pretreatment still retained a large amount of resin convexity regardless of whether it had undergone thermocycling, and there was a complete mixed layer around the resin convexity (Fig. 6A). These results indicate that OX-DAA can effectively delay aging of the resin–dentin bonding interface and maintain the integrity of the bonding interface. OX-DAA contributes to the integrity of the bonding interface in several aspects. First, OX-DAA effectively prevented the degradation of collagen fibers exposed at the bonding interface. The results showed that the penetration depth of the adhesive was not completely consistent with the demineralization depth of the dentin in either the etching-bonding system or the self-etching system, and the bottom of the mixed layer formed a weak layer composed of collagen fibers that were not wrapped by the adhesive resin. As water accumulates at the bonding interface, endogenous proteases in the mouth and teeth accelerate the degradation of the exposed collagen fibers, and eventually the resin or prostheses fall off [4]. OX-DAA can covalently cross-link with collagen fibers to maintain the stability of the collagen triple-helix, thereby preventing collagenase from binding to the enzyme-binding site and effectively preventing the degradation of collagen fibers. We verified the integrity of the bonding interface through a nano-microleakage experiment (Ammoniacal Silver Stain and Double Fluorescence). The experimental results showed that compared with the untreated group, there was no microleakage in the bonding interface before and after OX-DAA treatment, and the integrity of the bonding interface was well maintained (Fig. 6B to D). In addition, OX-DAA can directly inhibit collagenase activity and prevent the degradation of collagen fibers. In situ zymography of collagenase activity at the bonding interface showed no enzyme activity at the bonding interface before and after OX-DAA treatment (Fig. 6C).

Second, OX-DAA reduces the hydrolysis of the bonding interface resin. Gaps at the bottom of the hybrid layer that are not completely penetrated by the adhesive resin can serve as pathways for water penetration or enzyme invasion (both external and internal). Over time, water at the bottom of the hybrid layer penetrated the adhesive resin. Owing to the hydrogen bonding of water in the hydrophilic region of the polymer chain of the resin, the adhesive resin expands and finally causes hydrolysis of the resin [30,39]. After the OX-DAA treatment at the bonding interface, the −C=C− at the end of the DAA molecule was opened and polymerized with the −C=C− of the resin, and the amide groups in the DAA molecule were introduced into the resin network. Because amides are more resistant to hydrolysis than lipids, the resin is more resistant to hydrolysis after introducing DAA molecules.

Therefore, OX-DAA can effectively delay aging of the resin–dentin bonding interface, improve the stability of the bonding interface, and maintain the bonding strength for a long time.

### OX-DAA promotes collagen mineralization

Although improving the anti-degradation of the exposed collagen matrix in the bottom layer of the hybrid zone may prolong the durability of the resin–dentin interface, remineralizing the demineralized dentin collagen fibers may be the fundamental solution to prevent the integrity loss of the hybrid layer because the mineralized collagen fibers cannot be degraded by collagenases, and the remineralized collagen fibers have stronger mechanic properties. DAA is a dopamine derivative that initiates the biomimetic mineralization pathway of polydopamine-assisted HA formation [40,41], thereby regulating collagen mineralization. Under alkaline conditions, the −C−OH group of the DAA molecule was converted to a −C=O group (Figs. S2 and S3). The results showed that OX-DAA-modified collagen had strong Ca^2+^ binding capability (Fig. 1F and G), which may be because the −C=O group can effectively provide lone-pair electrons for coordination with *P* vacant orbitals of calcium ions, thus achieving chemical stability [42]. Therefore, OX-DAA-modified collagen showed improved collagen mineralizing activity.

In this study, OX-DAA cross-linked with collagen might have been formed by the Schiff base reaction, and the cross-linking mechanism was the same as that in natural collagen [43,44]. Biomimetic remineralization of dentin in living organisms requires 2 different kinds of dentin-like phosphoproteins: one is a chelator that controls amorphous precursor (ACP) aggregation; the other is a promoter that promotes the deposition of HA at specific sites of collagen, thus allowing mineral precursors to form nuclei at specific sites [45–47]. In this study, polyacrylic acid (PAA) was used as a polyanionic electrolyte to simulate the negative charge characteristics of noncollagen proteins, to stabilize ACP in the nanoprecursor state, and to prevent fluid ACP from aggregating into larger particles. OX-DAA molecules on the collagen surface guide ACP nanoprecursor to dentin collagen and nucleate and grow at specific sites to form “bottom-up” remineralized dentin with a similar morphological structure to biomineralized dentin [48].

Collagen mineralization includes both intrafibrillar and interfibrillar mineralization. In the experiment, OX-DAA-collagen rapidly completed intrafibrillar mineralization, whereas untreated collagen could not achieve intrafibrillar mineralization (Fig. 2A). There are 2 reasons for this discrepancy. First, the noncollagen protein analog PAA is used, and second, DAA modifies collagen. PAA is a polyanionic electrolyte containing a large number of –COOH groups that can chelate Ca^2+^ and stabilize calcium and phosphorus in a supersaturated solution to form ACP. Therefore, PAA can inhibit calcium-phosphorus nucleation to form HA, which is a mineralization inhibitor. The PAA (molecular weight: 3,000) used in this study is less than 6 kDa, and according to the size exclusion theory, it can freely enter and exit the collagen fibers, thus inhibiting their mineralization [49,50]. The main residues in untreated collagen molecules are glycine, proline, and HYP [51], which are neutral and inert during biomineralization [52]. Therefore, untreated collagen could not guide PAA-ACP to the collagen surface, and the PAA-ACP nanoparticles were uniformly distributed around the collagen. For OX-DAA-collagen, the quinones of OX-DAA molecules had a strong ability to attract calcium ions, so we could see the pool of mineralized precursors formed by the aggregation of PAA-ACP near collagen (Figs. 2B and 8CI and Fig. S7). Then, the PAA-ACP on the collagen surface enters the pore region inside the collagen fiber by capillary force, and the amorphous precursor begins to solidify and crystallize, forming a thermodynamically more stable crystal phase, and finally mineralizing the fiber (Fig. 8CII). The leading theory of collagen fiber mineralization is the polymer-induced liquid precursor theory [28,53]. HRTEM and SAED diffraction showed that the *c*-axis orientation of HA followed the coalignment of collagen fibers along the long axis (Fig. 2C). This is determined by the unique pore structure of the collagen fibers. The limitation in these pores and the anisotropic growth of HA determine the orientation of HA crystals within collagen fibrils [54].

When intrafibrillar mineralization is completed, the arrangement of HA microcrystals in the fiber is no longer confined to the inside of the collagen fibers, growing on the surface of the collagen fibers or between the 2 fibers, thus forming interfibrillar mineralization. In the untreated collagen surface, HA grew randomly and was disordered on the collagen surface due to the lack of ACP binding sites (Fig. 3A). The untreated collagen could not achieve intrafibrillar mineralization and had random and disordered deposition of HA on the surface of self-assembled collagen or demineralized dentin collagen after a period of time because of the lack of binding sites for ACP (Figs. 3A and 4C and D). OX-DAA molecules can guide ACP to the surface of collagen, nucleate, and grow at specific sites (Fig. 8DIII). The presence of OX-DAA reduced the potential energy of HA crystal nucleation on the collagen surface. Surface HA grew along the collagen fiber, and the *c*-axis orientation of HA followed the coalignment of collagen fibers along the long axis (Fig. 8DIII). In both self-assembled collagen fibers and naturally demineralized dentin collagen fibers, the growth of HA along collagen fibers was observed after OX-DAA treatment, and the c-axis of HA crystals was parallel to the c-axis of collagen fibers (Fig. 4E and F). The arrangement of HA crystals on the surface of collagen fibers is the same as that of the interfibrillar mineralization in organisms. Therefore, OX-DAA-treated collagen fibers can mineralize collagen fibers in vitro (both intrafibrillar and interfibrillar mineralization). The interfibrillar mineralization morphology of self-assembled collagen fibers, especially the interfibrillar remineralization morphology of demineralized dentin collagen fibers, has not been realized in other studies. Mineralized collagen fibers, the basic structural units of bone tissue, are composed of both intrafibrillar and interfibrillar mineralization [29,30]. The interfibrillar mineralization accounts for 75% of the total mineral content, whereas the intrafibrillar mineralization accounts for a relatively small proportion [42]. Mineralized collagen fibers are the main source of strength and fracture resistance in bone tissue and are also the focus of research in the field of biomimetic mineralization [55,56]. The realization of in vivo collagen fiber mineralization (including intrafibrillar and interfibrillar mineralization) has an enormous influence on the study of the mechanism of collagen fiber mineralization and the development of bone repair materials in tissue engineering.

The meaning of OX-DAA-treated collagen in realizing collagen fiber mineralization in vitro is beyond doubt. If the remineralization of the bonding interface, demineralized collagen fiber, can be realized in vitro, and even in vivo, it is the key to achieving resin–dentin permanent repair. To study the effect of OX-DAA on the bonding mineralization of the resin–dentin interface, we placed the resin–dentin bonding samples in mineralization solution containing PAA to simulate the mineralization environment after dental resin filling and restoration in vivo. There are 2 sources of calcium and phosphorus for the demineralized collagen fibers at the resin–dentin interface: one is through the penetration of the interface, and the other is through the fluid in the dentin tubules. Our experimental results showed that after 4 weeks of remineralization, only the collagen fibers demineralized at the bonding interface with OX-DAA as a primer could be remineralized, and the demineralized dentin around the dentin tubules was highly mineralized (Fig. 7E and Fig. S8). This indicates that the source of calcium and phosphorus in dentin tubules plays an important role in the remineralization of the bonding interface, which is consistent with the fact that dentin tubule fluid can be used as a source of calcium and phosphorus in organisms. Therefore, in vitro experiments showed that OX-DAA, as a primer, achieved the remineralization bonding interface of demineralized collagen in vivo. In this experiment, we selected rats as the in vivo study model for resin–dentin bonding interface remineralization. Considering the operational sensitivity of resin–dentin bonding technology and the degree of tooth abrasion in rats, we selected rat lower anterior teeth as experimental subjects. At the same time, to prevent the influence of individual differences in rats on the remineralization of the bonding interface, we used the left and right self-controls of the lower anterior teeth. In vivo, the sources of calcium and phosphate for remineralization of resin–dentin bonding interface mainly came from fluids of dentinal tubules or saliva where the resin–dentin bonding interface exposed to oral cavity. The time of this experiment was originally set at more than 2 weeks, but due to the rapid tooth abrasion of rats, after 12 d of adhesive resin filling, the lower anterior teeth of rats wore out near the bonding interface. Therefore, the experiment was terminated. Although the mineralization effect was obviously different between the in vivo and in vitro results, the demineralized bonding interface after OX-DAA treatment showed partial mineralization compared with the control group in vivo (Fig. 7G). There are several reasons for the great difference between the in vitro and in vivo remineralization of the bonded interfaces. First, the bonding interface in the rats was not remineralized for sufficient time. If the in vivo bonding interface is given sufficient time to mineralize, it may remineralize as well as in vitro. Second, the calcium and phosphorus sources for remineralization of the bonding interface in vitro are more direct and sufficient. In the in vitro experiments, resin–dentin bonded samples were cut to a size of 8 mm (length) × 8 mm (width) × 2 mm (thickness) and immersed in a mineralization solution containing PAA. The bonding interface and dentin tubules of the samples were filled with calcium and phosphorus solution. Compared with in vivo conditions, where teeth are not always immersed in saliva, and calcium and phosphate ions cannot be delivered to the binding interface, the concentrations of calcium and phosphorus in saliva and dentin tubule fluid were much lower than those in the in vitro mineralized fluid. In vivo, represents a complex environment that is influenced by many factors, such as cells and proteins. Therefore, complete remineralization of the bonding interface may take longer in vivo than in vitro. To the best of our knowledge, this is the first report of resin–dentin interface mineralization in vivo.

## Conclusion

OX-DAA can inhibit the activity of collagenase and cross-link collagen fibers to improve the anti-enzymatic hydrolysis ability of collagen fibers as well as induce intrafibrillar and interfibrillar collagen mineralization in vitro. As a primer used in the etch-rinse tooth adhesive system, OX-DAA can improve the durability and integrity of the bonding interface by degrading the exposed collagen matrix and inducing mineralization at the interface in vitro and in vivo. OX-DAA has shown potential as a primer for enhancing dentin durability; as such, it is optimal to use a 5% OX-DAA ethanol solution for treating the etched dentin surface for 30 s as a primer for the etch-rinse tooth adhesive system.

## Materials and Methods

### DAA and OX-DAA modify reconstituted collagen

#### Preparation of the DAA and OX-DAA solutions

DAA was freshly dissolved in anhydrous ethanol, and the pH value of the solution was adjusted to 5 with 6 M HCl to obtain unoxidized DAA. To oxidize DAA (OX-DAA), the pH value of the DAA solution was adjusted to 9 with 6.0 M NaOH and maintained for 24 h.

#### Preparation of the single-layer collagen fibrils and collagen membranes

The collagen stock solution was added to the assembling buffer solution (50 mM glycine, 200 mM KCl, pH 9.2) to obtain a 50 μg ml^−1^ collagen dilution solution and incubated at 37 °C for 12 h. Collagen membranes were prepared using a collagen dilution solution with a vacuum filtration setup (pressure 0.1 MPa) and filter paper.

#### Preparation of the DAA-collagen and OX-DAA-collagen

The collagen membranes were immersed in unoxidized DAA or oxidized DAA solution (concentrations 1, 5, and 10 wt%) for a certain duration (30 s, 60 s, 120 s, and 24 h). The collagen membranes were then rinsed repeatedly with anhydrous ethanol to remove uncombined cross-linkers. Finally, unoxidized DAA-modified collagen membranes (DAA-collagen) and oxidized DAA-modified collagen membranes (OX-DAA-collagen) were obtained using a critical point dryer (K850, Emitech Ltd., UK).

### Characterization of DAA and OX-DAA-modified collagen

The collagen membrane, DAA, and OX-DAA-modified collagen membranes were characterized by ATR-FTIR spectroscopy (Nicolet iS50, Thermo Scientific, USA) and a solid-surface zeta potentiometer (SurPASS, Anton Paar, Austria) (*n* = 3). The degree of cross-linking (the percentage of free amine groups in the collagen after DAA and OX-DAA cross-linking compared with un-cross-linked collagen) was determined using the ninhydrin assay to evaluate the cross-linking efficiency of DAA and OX-DAA [35].

### Characterization of DAA/OX-DAA properties

Collagen enzymolysis was detected in order to evaluate the antihydrolysis ability of the collagen membrane using the HYP content assay kit, as described by an ultraviolet-visible spectrophotometer (UV-1800, Shimadzu, Japan). Collagenase activity was tested using a collagenase activity colorimetric assay kit, as described by the absorbance kinetics method. Laser confocal microscopy (CLSM) and XPS were used to evaluate the calcium-binding ability of collagen and OX-DAA-collagen. Evaluation of OX-DAA induce collagen mineralization was performed by TEM (L 120C G2, Thermo Scientific Talos, USA) and FE-SEM (Gemini 500, Zeiss, Germany). The quality of the resin–dentin bonding interface was evaluated by μTBS (AGS-X, Shimadzu, Japan), ammoniacal silver nitrate staining, double fluorescence technique, in situ zymography, and bonding interface remineralization.

Detailed materials and methods can be found in the Supplementary Materials.

## Data Availability

All data needed of this study are available in the article and its supplementary information files.

## Supplementary Materials

Supplementary 1 Detailed materials and methods can be found in supplementary materials. Table S1. The parameters of ATR-FTIR spectra of collagen treated by different concentrations of DAA and OX-DAA. Figure S1. The chemical formula of N-2-(3,4-dihydroxylphenyl) acrylamide (DAA) molecule. Figure S2. 13C NMR analysis of oxidized DAA structures. Figure S3. Mass spectrometric analysis of DAA before and after oxidation. Figure S4. TEM characterization of self-assembled single-layer collagen. Figure S5. DAA molecules polymerized among and influence on adhesive polymerization. Figure S6. In vitro and in vivo resin–dentin interface remineralization observed by TEM. Figure S7. TEM characterization of OX-collagen collagen mineralization for 1 d. Figure S8. In vitro resin–dentin interface remineralization observed by TEM.
