# Supplementary material for: Oxidized Dopamine Acrylamide Primer to Achieve Durable Resin–Dentin Bonding
Source: Research (Wash D C). 2023 Apr 3;6:0101. doi: 10.34133/research.0101 (PMC10079285; doi:10.34133/research.0101)
Supplement: Supplementary 1 — Detailed materials and methods can be found in supplementary materials. Table S1. The parameters of ATR-FTIR spectra of collagen treated by different concentrations of DAA and OX-DAA. Figure S1. The chemical formula of N-2-(3,4-dihydroxylphenyl) acrylamide (DAA) molecule. Figure S2. 13C NMR analysis of oxidized DAA structures. Figure S3. Mass spectrometric analysis of DAA before and after oxidation. Figure S4. TEM characterization of self-assembled single-layer collagen. Figure S5. DAA molecules polymerized among and influence on adhesive polymerization. Figure S6. In vitro and in vivo resin–dentin interface remineralization observed by TEM. Figure S7. TEM characterization of OX-collagen collagen mineralization for 1 d. Figure S8. In vitro resin–dentin interface remineralization observed by TEM. [file research.0101.f1.docx]

**Supplementary Material**

**Oxidized Dopamine Acrylamide Primer to** **Achieve Durable Resin–Dentin Bonding**

Leping Wu ^1,^ †, Hui Shao ^1, 2,^ †, Yang Tao ^1^, Jingya Wu ^1^, Xinhui Wang ^1^, Qiufeng Nian ^1^, Shunli Zheng ^1^, Chris Ying Cao ^1^, Yuancong Zhao ^3^, Zheng Zhou ^4^, Hai Ming Wong ^5^, Quan-Li Li ^1,^*

*^1^ Key Lab. of Oral Diseases Research of Anhui Province, College & Hospital of Stomatology, Anhui Medical University, 81 Meishan Road, Hefei, 230032, China.*

*^2^* *The Affiliated Jiangning Hospital with Nanjing Medical University, Nanjing, Jiangsu, 211100, China.*

*﻿**^3^ Key Lab. of Advanced Technology for Materials of Education Ministry, School of Materials Science and Engineering, Southwest Jiaotong University, Chengdu 610031, China.*

*^4^* ﻿*School of Dentistry, University of Detroit Mercy, Detroit, Michigan 48208-2576, United States.*

*^5^* ﻿*﻿Faculty of Dentistry, The University of Hong Kong, 34 Hospital Road, The Prince Philip Dental Hospital, Hong Kong 999077, China.*

Correspondence should be addressed to Quan-Li Li; [ql-li@126.com](mailto:ql-li@126.com)

† These authors contributed equally to this work**.**

﻿

**1.** **MATERIALS AND METHODS**

**1.1. Materials**

Collagen-I stock solution (Corning Incorporated, NY, USA) was extracted from the rat tail tendon. Highly purified collagenase type I (Sigma-Aldrich, St. Louis, MO, USA) obtained from Clostridium histolyticum. A micro hydroxyproline (HYP) content assay kit (Solarbio, Beijing, China) and collagenase activity colorimetric assay kit (Biovision, San Francisco, USA) were used to measure resistance to enzymatic hydrolysis. Tooth adhesive of Adper^TM^ Single Bond 2 (3M, St. Paul, MN) and Filtek™ P60 post-tooth composite resin (3M, St. Paul, MN) was used for dentin bonding experiments. Poly (acrylic acid) (PAA, Mw: 3 kDa, Aladdin, Shanghai, China) were used to stabilize a supersaturated calcium phosphate (CaP) mineralization medium. Other chemicals used were CaCl_2_·2H_2_O (Sigma-Aldrich, St. Louis, MO, USA), Na_2_HPO_4_ (Sigma-Aldrich, St. Louis, MO, USA), Glycine (Aladdin, Shanghai, China), KCl (Aladdin, Shanghai, China). DAA was synthesized by our group (Fig. S1) [1].

**1.2.** **DAA and OX-DAA Modify** **Reconstituted Collagen**

**Reconstituted Collagen:** The method of collagen self-assembly was carried out as the previous work [2]. Collagen-I stock solution were added into the assembling buffer solutions (50 mM glycine, 200 mM KCl, pH 9.2) to obtain 50 μg mL^−1^ collagen-I solution and ﻿incubated at room temperature for 2 h and incubated at 37 °C for 12 hrs. The collagen membranes were prepared using the abovementioned self-assembled collagen-I solution with a vacuum filtration setup (pressure of 0.1 MPa) and qualitative filter paper and then rinsed with deionized water.

**Prepare DAA and OX-DAA Solution:** DAA was dissolved in anhydrous ethanol freshly, and the pH value of the solution was adjusted to 5 with 6 M HCl to get the un-oxidized DAA. To oxidize DAA (OX-DAA), the pH value of the DAA solution was adjusted to 9 with 6.0 M NaOH, and kept for 24 hrs.

**Characterize DAA and OX-DAA:** ^13^C Nuclear Magnetic Resonance (NMR) of DAA and its oxidized product were carried out by Bruker AVANCE III 400. The pristine DAA was dissolved in deuterated methanol (CD_3_OD) and detected immediately (denoted by “0 min”), then adjust the solution to pH=9 by NaOH to initiate the oxidation. The oxidized DAA (OX-DAA) was detected 30 minutes (30 min) and 24 hours (24 h) later, respectively. Mass spectra (MS, Sciex API 150EX LC/MS) analysis was carried out to monitor the changes of molecular weight of DAA and its oxidized product. Mass spectra were recorded in the m/z range of 180-260. The DAA was dissolved in anhydrous ethanol at a concentration of 0.1 mg/ml, followed by adjusting the solution to pH=9 by NaOH and oxidized for 24 hours. The pristine DAA and oxidized DAA were measured, respectively.

**Prepare DAA and OX-DAA Membranes:** The 24 mg collagen membranes were immersed in the 20 mL unoxidized DAA or oxidized DAA solution (concentrations 1, 5, and 10 wt%, respectively) for certain duration (30 s, 60 s, 120 s and 24 hrs, respectively). Then the collagen membranes were rinsed repeatedly with anhydrous ethanol and water to remove the excess cross-linker. ﻿Finally, the unoxidized DAA modified collagen membranes (DAA-Collagen) and oxidized DAA modified collagen membranes (OX-DAA-Collagen) were obtained via using a critical point dryer (K850, Emitech Ltd., UK).

﻿ **Characterize DAA and OX-DAA Membranes.**

**Attenuated Total Reflection−Fourier Transform Infrared Spectroscopy (Thermo Scientific, Nicolet iS50, USA).** The collagen and OX-DAA-Collagen membranes were evaluated by attenuated total reflection−Fourier transform infrared (ATR−FTIR) spectroscopy from 4000 to 500 cm^−1^ at 4 cm^−1^ resolution in 20 scans.

**﻿Solid Surface Zeta Potential (SurPASS, Anton Paar, Austria):** At room temperature, four pieces of 10 × 20 mm collagen and OX-DAA-Collagen membranes were adhered onto four sample tables, and the functional layers of the two pieces of samples were placed into the sample pool. The electrodes were connected, and an electrolyte (1 mM potassium chloride solution, pH = 7) was injected into the sample end for washing. The washing pressure was 100 mbar. The height of the sample cell channel was adjusted until the electrolyte flow rate was 100 ± 20 mL/min. The test pressure was 300 mbar, and the samples were tested two times in the left and right directions. Finally, the average value was calculated. The tests were repeated four times for each group of samples (n = 5).

**UV−visible Spectrophotometer (UV-1800, Shimadzu, Japan):** The cross-linking degree of the cross-linked collagen membrane was measured by the ninhydrin (2,2-dihydroxy-1,3-indanedione) method measuring free amino groups. The 300 mg collagen and OX-DAA-Collagen membranes were heated with the ninhydrin coloration solution (85 mg of ninhydrin and 15 mg of hydrindantin dissolved in 10 mL of 2-methoxyethanol) at 100 °C for 15 min. Optical absorbance was then recorded with a UV−visible spectrophotometer (UV-1800, Shimadzu, Japan) at 570 nm. Glycine at various known concentrations was used to calculate the standard curve [1]. The cross-linking degree was calculated as follows:

**Cross-linking degree (%) = 100 (M_0_ - M_t_) / M_0_**

where M_0_ is the amount of the free amino groups in collagen films before cross-linking and M_t_ is the amount of the free amino groups in the collagen films after cross-linking (n = 5).

**1.3. Anti-enzymolysis of DAA/OX-DAA-Collagen** **and DAA/OX-DAA Inhibition** **of Collagenase Activity**

**Anti-enzymolysis of DAA/OX-DAA-Collagen:** A certain amount of unmodified collagen membranes, and DAA/OX-DAA-Collagen membranes as above description treated with different DAA/OX-DAA concentration and different time, were immersed into 1 mL of 100 U/mL collagenase buffer solution containing (0.36 mM CaCl_2_, 0.02% NaN_3_ and 50 mM Hepes buffer, pH＝7.4）for 24 hrs (37ºC). Then the special product of hydroxyproline (HYP) of collagen enzymolysis was detected to evaluate the anti-hydrolysis ability of collagen membrane using HYP content assay kit as described by UV-visible spectrophotometer (UV-1800, Shimadzu, Japan) at 560 nm to record the absorbance [1, 3] (n= 5).

**DAA/OX-DAA** **Inhibition of** **Collagenase Activity:** This assay was performed in a 96-well plate and divided into four groups. Negative control: 10 µL of the provided collagenase (0.35 U/mL). Positive control: 2 µL of inhibitor (1,10-phenanthroline) + 10 µL of the provided collagenase. Experimental group: 2 µL of 5% OX-DAA (pH = 9) +10 µL of the provided collagenase，or 2 µL of 5% OX-DAA (pH = 5, OX-DAA regulates pH to 5) + 10 µL of the provided collagenase, The solution volumes in each well of all the groups were adjusted to 100 µL with the provided collagenase assay buffer and then incubated at room temperature for 10 min. a well only containing 100 µL of the provided collagenase assay buffer was used as reagent background for the test. Then, collagenase activity was tested by the collagenase activity colorimetric assay kit as described by the absorbance kinetics method at OD 345 nm (n =5) [3].

**1.4 Evaluation of** **Calcium-binding Ability of Collagen and OX-DAA-Collagen**

**Confocal laser scanning microscope (CLSM; LSM-880, Carl Zeiss, USA).**

The collagen and OX-DAA-Collagen membranes were soaked in CaCl_2_ (9.5 mM) containing calcein (1 μM) at room temperature for 4 h, then fully washed to remove unbound Ca^2+^ and dried. The binded calcium of the collagen and OX-DAA-Collagen membranes was labeled with calcein. Fluorescence images were obtained using an inverted confocal laser scanning microscope. Specimens were illuminated with a 488-nm laser. All images were captured and analyzed with an image analysis software (ZEN Blue Lite2_3).

**X-ray photoelectron spectroscopy (XPS, Thermo Scientific ESCALAB 250Xi).** The collagen and OX-DAA-Collagen membranes were soaked in CaCl_2_ (9.5 mM) at room temperature for 4 h, then fully washed to remove unbound Ca^2+^ and dried. **﻿**Chemical composition of the collagen and OX-DAA-Collagen membranes surfaces was confirmed by XPS survey scans collected between 0-1,100 eV binding energies, with detailed scans of C1s, O1s, N1s and Ca2p electrons performed in triplicate for element composition evaluation.

**1.5. Evaluation of OX-DAA Mineralizing Collagen**

**1.5.1. Preparation of Mineralizing Solutions.**

The mineralizing solution contained 1.67 mM of CaCl_2_, 9.5 mM of Na_2_HPO_4_, 150 mM of NaCl, 75 μg/mL of PAA, and 0.02% (w/v) NaN_3_. In preparing the solution, a calcium ion solution (3.34 mM CaCl_2_) was added to the beaker, and 10 mg/mL of the PAA stock solution was added to the calcium solution and mixed thoroughly. Then, an equal volume of the phosphate ion solution (19 mM Na_2_HPO_4_ and 300 mM NaCl) was added to the above-mentioned Ca^2+^mixed solution under agitation. Adding 0.02% (w/v) NaN_3_ to prevent bacterial growth [2].

**1.5.2. Preparation of** **Nickel Grids Collagen and Demineralized Dentin Collagen.**

**Reconstitution of** **single layer collagen﻿ on nickel grids.** Collagen-I stock solution were dripped into the assembling buffer solutions to obtain 50 μg mL^−1^ collagen-I solution and the collagen-I solution (3 μL) was dropped onto a ﻿nickel grids. The ﻿nickel grids were incubated at 37 °C for 12 h.

**Demineralized dentin collagen.** Extracted healthy human third molars were collected from the patients who gave their consent under a protocol approved by the Ethical Committee of the Anhui Medical University, China (No. 2019 0234). Dentine slices with dimensions of 4 (length) × 4 (width) × 1.5 (depth) mm were prepared, all dentine slices surfaces were polished with 400-, 600-, 800-, 1200-, and 2000-grit silicon carbide papers under running water and then ultrasonically cleaned with anhydrous acetone, anhydrous ethyl alcohol, and deionized water alternatively (three times for 10 min each). The surface demineralized dentine collagen matrix layer was produced by etching with 35% phosphoric acid at 25 °C for 20 s. Subsequently, all of the slices were washed with excess triple distilled water.

As above mentioned, the nickel grids single layer collagen and the demineralized dentin collagen matrix were modified with OX-DAA as above described for 24 hrs.

**Collagen Mineralization.** The nickel grids collagen, collagen membrane and demineralized dentin slice were immersed into 200 ml of the mineralizing solutions at 37 °C, and the samples were randomly collected at speciﬁed intervals. The mineralized solution was changed at 48 hrs intervals.

**Transmission Electron Microscopy** **(TEM,** **L 120C G2, Thermoscientific Talos, USA):** The untreated collagen and OX-DAA-Collagen on nickel grids before and after mineralization were examined by TEM to observe the intrafibrillar mineralization. Selected area electron diﬀraction (SAED) was performed to identify the crystallinity of the mineralized crystals and their respective orientation. The untreated collagen and OX-DAA-Collagen mineralized collagen membranes were immersed in propylene oxide and embedded in epoxy resin to take ultramicrotomy for TEM examination.

**Field Emission Scanning Electron Microscopy (FE-SEM, Gemini 500, Zeiss, Germany):** The mineralized collagen membranes and remineralized dentin were sprayed on a gold−palladium sputtering coater (Hitachi, Japan E-1010) at 15 mA for 40 s and then observed by ﻿FE-SEM under a 5 kV acceleration voltage to observe the interfibrillar mineralization.

**X-ray diffraction (XRD, Rigaku America, Woodlands, TX, USA):** The mineralized collagen membranes (collagen and OX-DAA-Collagen) were detected via XRD under the conditions of Cu Kα ray, voltage of 30 kV, current of 40 mA, scanning angle of 2θ = 10−60°, and scanning speed of 6°/min. The MDI Jade 6.5 software was used to analyze the scanning results, which were then compared with the HAP card for the powder diffraction standard.

﻿**Thermogravimetric Analysis (Q5000IR, TA Instruments, USA):** TGA to assess mineral content. Approximately 10 mg of the mineralized collagen membranes (collagen and OX-DAA-Collagen) was placed into the Al_2_O_3_ pan and heated at the rate of 30 °C/min to 800 °C in air. The data were analyzed using the Universal Analysis 2000 software and expressed as weight vs temperature. Each sample group was analyzed five times (n = 5).

**1.6.** **Evaluation of Inﬂuence of DAA and OX-DAA on the Polymerization of Adhesive**

In order to prove that C=C of DAA itself could be polymerize, light-initiator of camphorquinone (CQ, 3 wt‰) was added into OX-DAA and DAA solutions. One drop of mixed solution was spread on the top plate of ATR-FTIR, and polymerized by light at wavelength of 380 - 515nm inducing for 120 s with a LED device which used for light-curing dental materials (Bluephase 20i, IvoclareVivadent, Schaan, Liechtenstein). After light-curing, the spectra of FTIR were obtained with 10 scans in the range of 1800–1500 cm^-1^ with a resolution of 4 cm^-1^. To evaluate the potential inﬂuence of remnant DAA on the polymerization of dental adhesive, 360 μL of dental adhesive (AdperTM Single Bond 2, 3M, St. Paul, MN) with 40 μL of DAA and OX-DAA were mixed respectively, and pipetted to a brown sample tube and fully oscillated for 30 s before test. Then the detection method is the same as the above method. The degree of –C=C– conversion (DC) was then calculated by the following equation:

$$\boldsymbol{DC=(1-}\frac{\mathbf{Absorbance}_{\boldsymbol{methacrylate}}^{\boldsymbol{cured}}\boldsymbol{/}\mathbf{Absorbance}_{\boldsymbol{phenyl}}^{\boldsymbol{cured}}}{\mathbf{Absorbance}_{\boldsymbol{methacrylate}}^{\boldsymbol{uncured}}\boldsymbol{/}\mathbf{Absorbance}_{\boldsymbol{phenyl}}^{\boldsymbol{uncured}}}\boldsymbol{)}\boldsymbol{\times}\boldsymbol{100\%}$$

The DC was assessed according to the changes in absorbance ratio between the peak at 1680 ~ 1620 cm^-1^ (methacrylate C=C stretching) and that at ~ 1608 cm^-1^ (phenyl C=C stretching) before and after light-curing [4].

**1.7. Evaluation of the Quality of Resin-Dentin Bonding Interface**

**1.7.1. Resin-Dentin Bonding Strength**

Forty intact human third molars were prepared for resin-dentin bonding samples, and randomly divided into two groups. Teeth was sectioned perpendicular to the long axis of tooth under water cooling to expose the mid-coronal dentin surface. The dentin surface was wet-polished (600-grit SiC paper) for 1 min, etched with phosphoric acid (Scotchbond Universal Etchant, 3 M ESPE, St. Paul, MN, USA) for 20 s, thoroughly rinsed with water spray, and blot dried to achieve a slightly moist surface. The 5 wt% OX-DAA solution were scrubbed onto the surface with disposable applicators for 30s to achieve a slightly moist surface (experimental groups). The control group was treated with the same solution un-containing OX-DAA of the experimental groups. Then, normal adhesion operation was carried out in accordance with the manufacturer's instruction. Namely, the Adper^TM^ Single Bond 2 was rubbed on the dentin surface circularly, and dried with compressed air, followed by light-curing at approximately 600 mW/cm^2^ radiation for 20 s. Five of 1-mm thick Filtek™ P60 resin composite layers were incrementally built up on the adhesive and polymerized for 20 s for each layer. The composite bonded teeth were stored in distilled water at 37 °C for 24 h. The bonded teeth were sectioned parallel to the long axis to yield three of 1 mm thick resin-dentin slabs for each tooth, and then slabs were sectioned into resin-dentin beams with dimensions of 1 mm × 1 mm × 8 mm for micro-tensile bond strength test [3]. The OX-DAA and control group resin-dentin beams were taken respectively, and each group was randomly divided into two sub-groups again, namely immediate group (only 24 h water storage at 37 °C) and aged group (5,000 times thermocycling, 5-55 °C, 15 s dwell time and 7 s transferring time). After the above treatment, the qualiﬁed samples were ﬁxed on the micro-tensile bond strength tester (μ TBS, AGS-X, Shimadzu, Japan) (n = 40) [5-7]. A universal testing machine was used to carry out micro-tensile test on the bonded specimen at the tensile rate of 1 mm/min parallel to the long axis of the specimen, and the maximum load (F) at the time of fracture was recorded. An electronic vernier caliper was used to record the length (L) and width (W) of the fracture interface, and the micro-tensile strength (MPa) = F / (L × W) was calculated according to the micro-tensile experimental formula. The result was analyzed by two-way ANOVA ﻿(Factors: OX-DAA and aging) followed by *post hoc Tukey's test* (α = 0.05) after validating the normality and homogeneity of the data.

**1.7.2. Failure mode analysis**

The fractured ﻿test specimen after μTBS test were collected and observed under fully automatic fluorescent stereo microscope (LEICA M205 FA, Germany), ﻿at 40X magnification to classify the type of ﻿fracture. The fracture types were classified as: adhesive (failure between the adhesive and dentin), cohesive in dentin (failure of the tooth substrate), cohesive in resin (failure of the composite resin), and mixed (adhesive and cohesive failure in resin) [8, 9]. ﻿The percentage of failure pattern in each group before and after aging was calculated, and the difference in the percentage was analyzed using Chi-Square test between the groups within each time point (α = 0.05).

**1.7.3.** **Morphological Characteristics of Resin-Dentin** **Bonding Interfaces.**

The OX-DAA and control group resin-dentin beans (immediate group and thermocycling aged group; n = 3) were immersed in 37% phosphoric acid and 2% hypochlorous acid for 1 week to remove all the dentin tissue, including the calcified dentin tissue and the demineralized dentin collagen matrix. The solution was changed every 12 h. The specimens were sprayed on a gold−palladium sputtering for SEM observation.

**1.7.4. ﻿Nanoleakage of** **Resin-Dentin** **Bonding Interface**

**Ammoniacal Silver Nitrate Stain.** The OX-DAA and control group resin-dentin slabs (immediate group and thermocycling aged group; n = 3) of an approximately 1-mm width area around the resin-dentin interface was left unprotected, while the rest part of the slabs was brushed with nail varnish for two layers. The specimens were soaked into 50 wt% ammoniacal silver nitrate solution for 24 hrs, and rinsed thoroughly in distilled water. After that, specimens were immersed in photo-developing solution for 8 h under a fluorescent light to reduce silver ions to metallic silver grains within voids along the bonded interface. After treatment, the specimens were rough polished using a series of SiC paper with 600-, 1200-, 2000-, and 3000-grit. The specimens were then ultrasonically cleaned, dried, sputter-coated with gold and observed via FE-SEM under backscattered mode.

**Double Fluorescence Technique.** Teeth was sectioned perpendicular to the long axis under water cooling to expose the mid-coronal dentin surface without exposing pulp chamber. The samples were randomly divided into OX-DAA and control group and each group were divided into immediate and thermocycling aged subgroups (n = 3) as above. Resin bonding of all groups were performed as mentioned above, except that the adhesive was premixed with 0.05 wt% rhodamine B (Solarbio, Beijing, China) to show red ﬂuorescence prior to adhesive application. Then, the thermocycling aged subgroups were subjected to thermocycling as described above. After that, all group samples were sectioned perpendicular to the long axis to expose the pule chamber, and the exposed pulp chamber was etched for 20 s with 37% phosphoric acid and then ﬁlled with 0.1 wt% aqueous ﬂuorescein solution (showing green ﬂuorescence) for 3 hrs (Solarbio, Beijing, China). The ﬂuorescein inﬁltrated from the pulp chamber through the dentinal tubules into the resin–dentin interface. Thereafter, specimens were copiously rinsed with water and treated in an ultrasonic water bath for 2 min. Each specimen was cut vertically to expose the resin–dentin interface and polished using an ascending grit of SiC abrasive papers. Then, the specimens were ultrasonically cleaned (5 min), and observed by confocal laser scanning microscope (CLSM; LSM-880, Carl Zeiss, USA).

**1.7.5.** **In Situ Zymography of the Resin-dentin Interface**

The surface containing resin-dentin interface of OX-DAA and control group resin-dentin slabs (immediate group and thermocycling aged group; n = 3) as above were wet polished, ultrasonically cleaned, and placed on glass slides. The 50 μL solution of quenched ﬂuorescein-conjugated gelatin reagent (E-12055, Molecular Probes, Eugene, OR, USA) was dropped on the surface containing the bonding interface of each slab. The slabs were incubated at 37 ^◦^C in the dark for 24 h in a humidity chamber. Green ﬂuorescence (λ_ex_ /λ_em_ = 488/530 nm) released after gelatin hydrolysis by the MMPs was imaged with a CLSM. The green ﬂuorescence detected within the resin-dentin interface indicates the activity of MMPs in situ.

**1.8. Cytocompatibility**

Sprague-Dawley (SD) bone marrow mesenchymal stem cells (SD-BMSCs) were obtained from SD rat (age: 3–4 weeks). Passage 3 cells were used in the study. The cell activity was evaluated using a CCK-8 assay (Dojindo Laboratories Inc., Kumamoto, Japan). The SD-BMSCs at a density of 1 × 10^4^ cells/ml were seeded in 24-well plates containing the untreated collagen and OX-DAA–Collagen membranes (n = 12). After the 1st, 3rd, 5th, and 7th days of the incubation period, using a CCK-8 assay (Dojindo Laboratories Inc., Kumamoto, Japan) to evaluate cell activity by MQX200 absorbance microplate reader (BioTek, USA) at 450 nm, and some of the sample was TRITC-phalloidin stained to evaluate cell morphology under inverted fluorescence microscopy (Axio Observer 3, ZEISS, Germany).

**1.9. Bonding interface Mineralization**

**1.9.1. Resin-Dentin Bonding Interface Mineralization** **in** **Vitro**

The resin-dentin bonding samples of the control group and OX-DAA group were cut into 2 mm thick resin-dentin slabs by parallel to 1.0 mm above and below the resin dentin bonding interface, and then were cut into 8 (length) × 8 (width) × 2 (thickness) mm resin-dentin beams, and wet polished, ultrasonically cleaned. The resin-dentin beams were immersed into 200 ml of the mineralizing solutions as above at 37 °C for 4 weeks. The mineralized solution was changed at 48 hrs intervals.

**1.9.2. Resin-Dentin Bonding interface Mineralization** **in Vivo**

﻿ Under isoflurane inhalation anesthesia, the Sprague–Dawley (SD) rat (3~4-week-old male) were randomly divided into 3 groups (n = 6). High Speed airturbine handpiece (PANA-MAX2, NSK, Japan) and carborundum ball drill (BR-49, MANI, Japan) were used to prepare a 1mm × 1.5 mm cavity defect with the depth up to dentine in the neck of the lower anterior teeth. ﻿For the first group, normal resin-dentin adhesion operation using a etch and rinsing adhesive system as above was performed in the tooth defect cavity, ﻿then SD rat were sacrificed immediately. ﻿For the control group, normal resin-dentin adhesion operation was performed, and SD rat were sacrificed ﻿after 12 days. For the OX-DAA group, except 5 wt% OX-DAA solution as a primer were scrubbed onto the defect surface with disposable applicators for 30s after acid-etching, all the adhesion operation was performed as control groups. SD rat were sacrificed ﻿after 12 days. The SD rat lower anterior cavity were retrieved for evaluation.

﻿Following retrieval in vitro/vivo resin-dentin samples, all the specimens were fixed in 2% glutaraldehyde, and specimen were rinsed three times with sodium cacodylate buffer. The specimen was dehydrated in an ascending ethanol series (50–100%), immersed in propylene oxide as a transitional medium and embedded in epoxy resin. For all specimens, 100 nm thick sections were prepared and examined using a TEM at 120 kV.

**2.0. Statistical Analysis**

| Groups | Parameters | | | | | |
| --- | --- | --- | --- | --- | --- | --- |
|  | Amide I (cm^-1^) | | | Amide II (cm^-1^) |  | Amide I (Abs) |
| Collagen | | 1634.15 |  | 1548.63 |  | 0.1791434 |
| DAA-Collagen (1%) | | 1634.26 |  | 1552.30 |  | 0.2268783 |
| DAA-Collagen (5%) | | 1634.68 |  | 1552.48 |  | 0.2562474 |
| DAA-Collagen (10%) | | 1652.02 |  | 1552.76 |  | 0.3207209 |
| OX-DAA-Collagen (1%) | | 1645.16 |  | 1552.46 |  | 0.2061102 |
| OX-DAA-Collagen (5%) | | 1645.16 |  | 1552.36 |  | 0.2540886 |
| OX-DAA-Collagen (10%) | | 1652.11 |  | 1552.62 |  | 0.3602736 |

Quantitative results are expressed as mean ± standard deviation. Statistical differences were analyzed by using the analysis of variance (ANOVA) followed by Tukey test, and a p-value < 0.05 was considered as statistically significant.

**2.** **RESULTS**

**Table S1.** The parameters of ATR-FTIR spectra of collagen treated by different concentration DAA and OX-DAA.

The parameters of ATR-FTIR spectra of collagen treated by different concentration DAA and OX-DAA were shown in **Table S1.** It could be observed that all the representative bands of collagen triple helix structure existed after DAA and OX-DAA treatment. There was no obvious change of the peak position except a minor shift of amide I band from 1634 to 1653 cm^-1^ and amide II band from 1548 to 1552 cm^-1^. In addition, the absorption peak intensity of amide I of collagen was slightly increased after cross-linking by DAA and OX-DAA (Each group collagen membrane was same thickness).

**Figure S1: The chemical formula of** **N-2-(3,4-dihydroxylphenyl) acrylamide (DAA) molecule.**


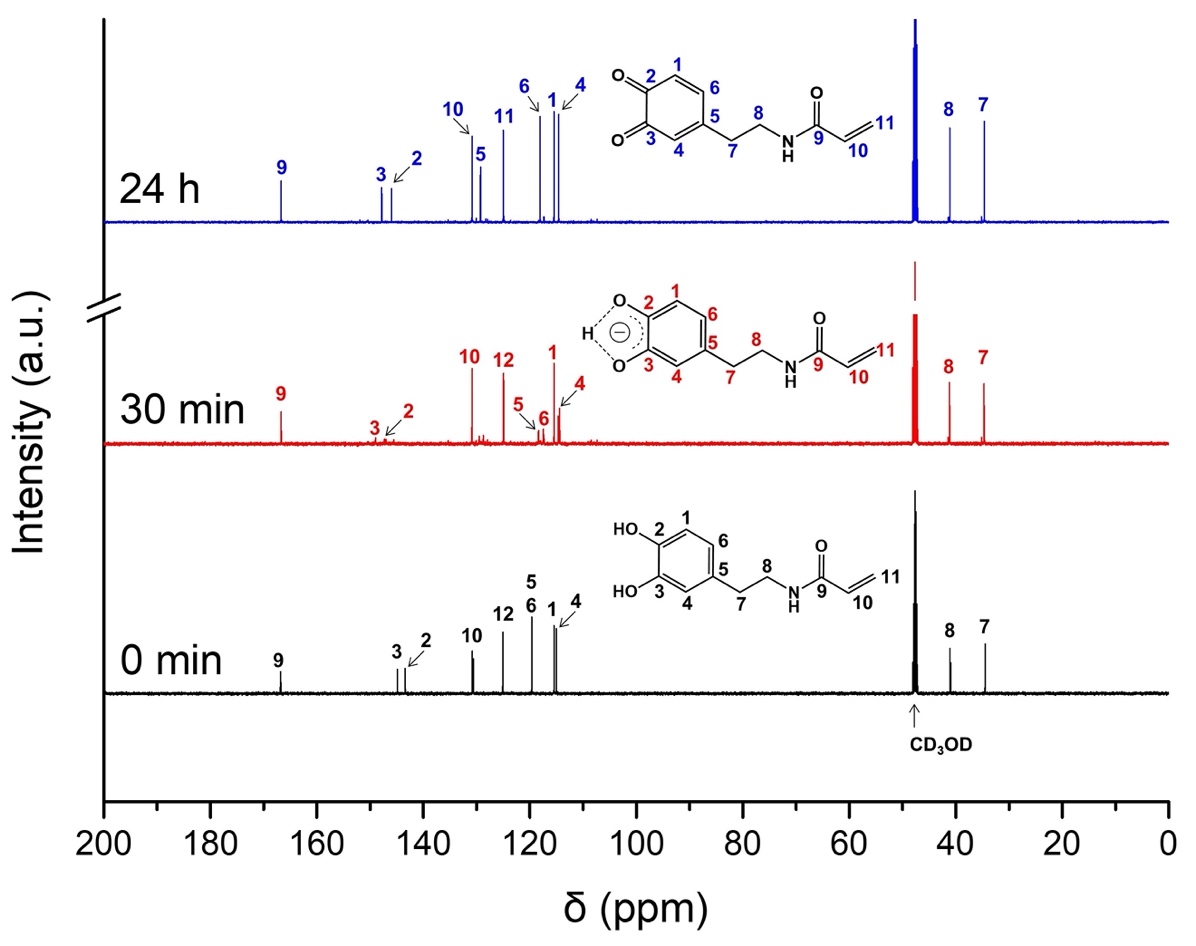


**Figure S2:** ^13^C NMR analysis of pristine and oxidized DAA.

For the pristine DAA, the peaks in the spectrum (0 min) assigned to each carbon atoms were shown in the Fig. S2. The peaks of sp^3^ hybridized carbon atoms (C7 and C8) located at high field (30-50 ppm), and sp^2^ hybridized carbon atoms (C1-C6, C10, C10 and C11) located in the low field (110-150 ppm). Specially, C9, C2 and C3 have high chemical shift (140-170 ppm) due to the deshielding effect by adjacent oxygen atoms.

At the beginning (30 min) of deprotonation of DAA by NaOH, one of the phenolic hydroxyl groups was deprotonated to form a conjugated negative ions, by which strengthened the deshielding effet of C2 and C3, leading to their shifting to lower field and weakened intensity, meanwhile C5 and C6 shifted toward higher field [10].

After 24 h of oxidation of DAA, C3 and C2 were shifted to lower field obviously when compared to the pristine DAA (0 min), indicating that the ionized catechol had been oxidized to quinone. In contrast, C1, C4, C10, and C11 did not show significant chemical shifts, and no new peaks were generated in high field, indicating that there was no polymerization between benzene rings or between olefinic bond (C10-C11) during the oxidation process.


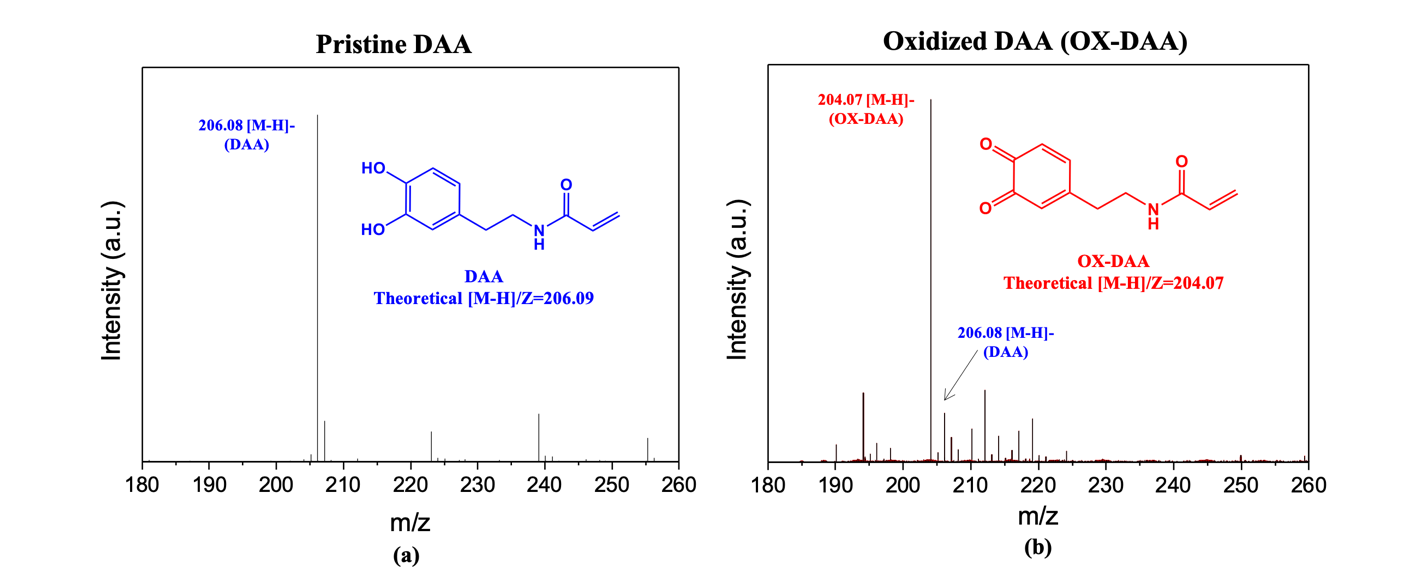


**Figure S3:** Mass spectrometric analysis of DAA (a) before and (b) after oxidation.

The molecular masses of DAA before and after oxidation were detected by mass spectroscopy (negative ions). The main m/z peak of pristine DAA ([M-H]-) was at 206.08, while that of oxidized DAA was at 204.07, which were consistent with the expected phenolic- and quinone-type DAA, respectively.

The ^13^C-NMR and MS results revealed that most of the DAA could convert to the quinone-type structure without unwanted side reaction after its oxidation in alkaline ethanol for 24 hours. This providing a solid foundation to support the grafting of the oxidized DAA to collagen through Schiff base reaction.


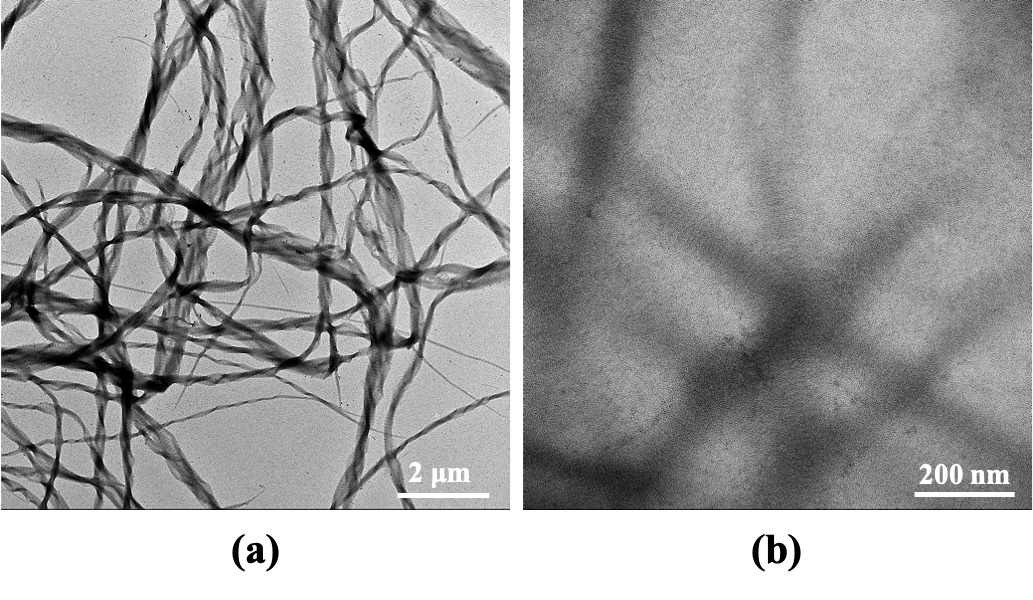


**Figure S4:** **TEM ﻿characterization of self-assembled ﻿single layer collagen.** (a) ﻿Unstained collagen fibrils. (b) ﻿Uranyl acetate-stained collagen fibrils. The results showed that the collagen fiber self-assembled well, and the 67 nm transverse lines were obvious.


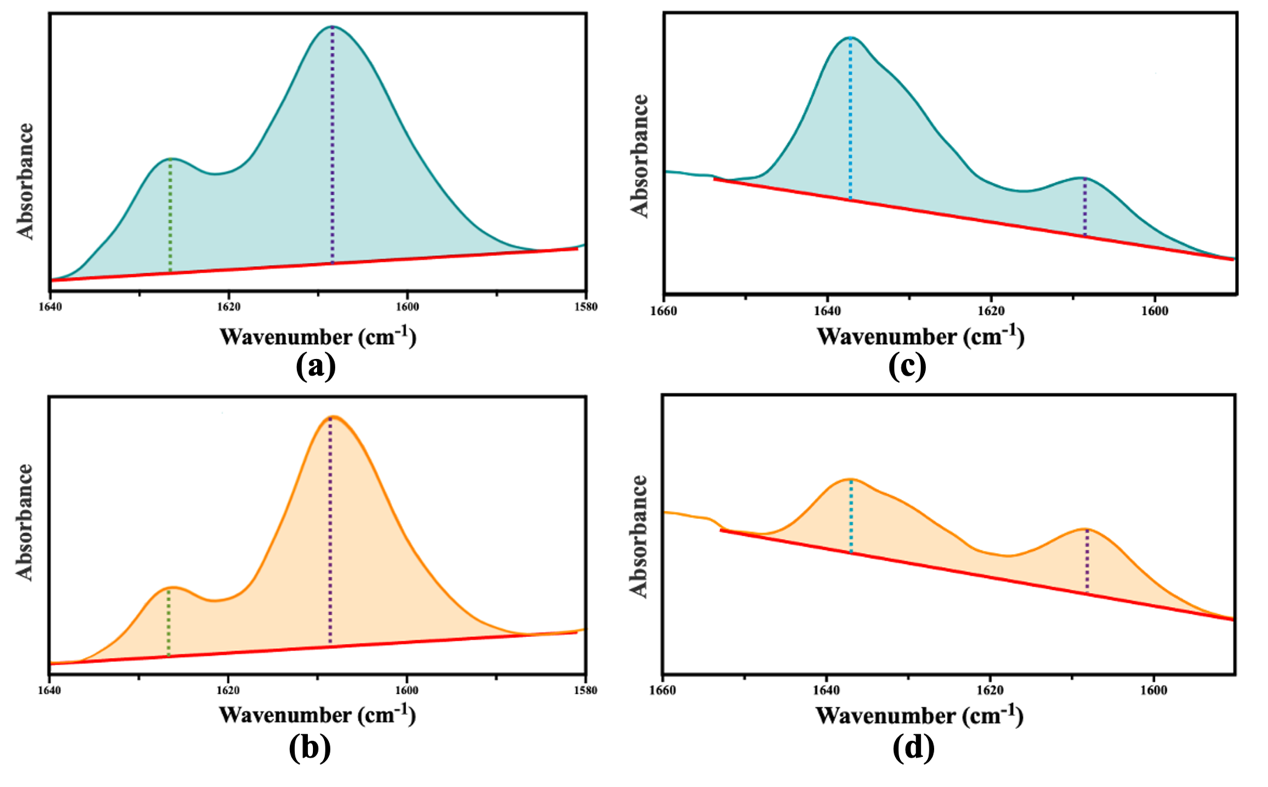


**Figure S5: DAA molecules polymerized among and inﬂuence on adhesive polymerization.** (a) ﻿Representative C=C peaks of ﻿FTIR spectra of DAA mixed camphorquinone before light-curing. (b) ﻿Representative C=C peaks of ﻿FTIR spectra of DAA mixed camphorquinone after light-curing. (c) ﻿Representative C=C peaks of ﻿FTIR spectra of DAA mixed commercial adhesive before light-curing. (d) ﻿Representative C=C peaks of ﻿FTIR spectra of DAA mixed commercial adhesive after light-curing.

After 120s photocuring, DAA molecules polymerized among each other, the ﻿degree of conversion of DAA molecule reached 40.60% (Figure 5S(a), (b)). After 30 seconds of light curing, the degree of conversion of DAA mixed commercial resin reached 57.60% (Figure 5S(c), (d)).


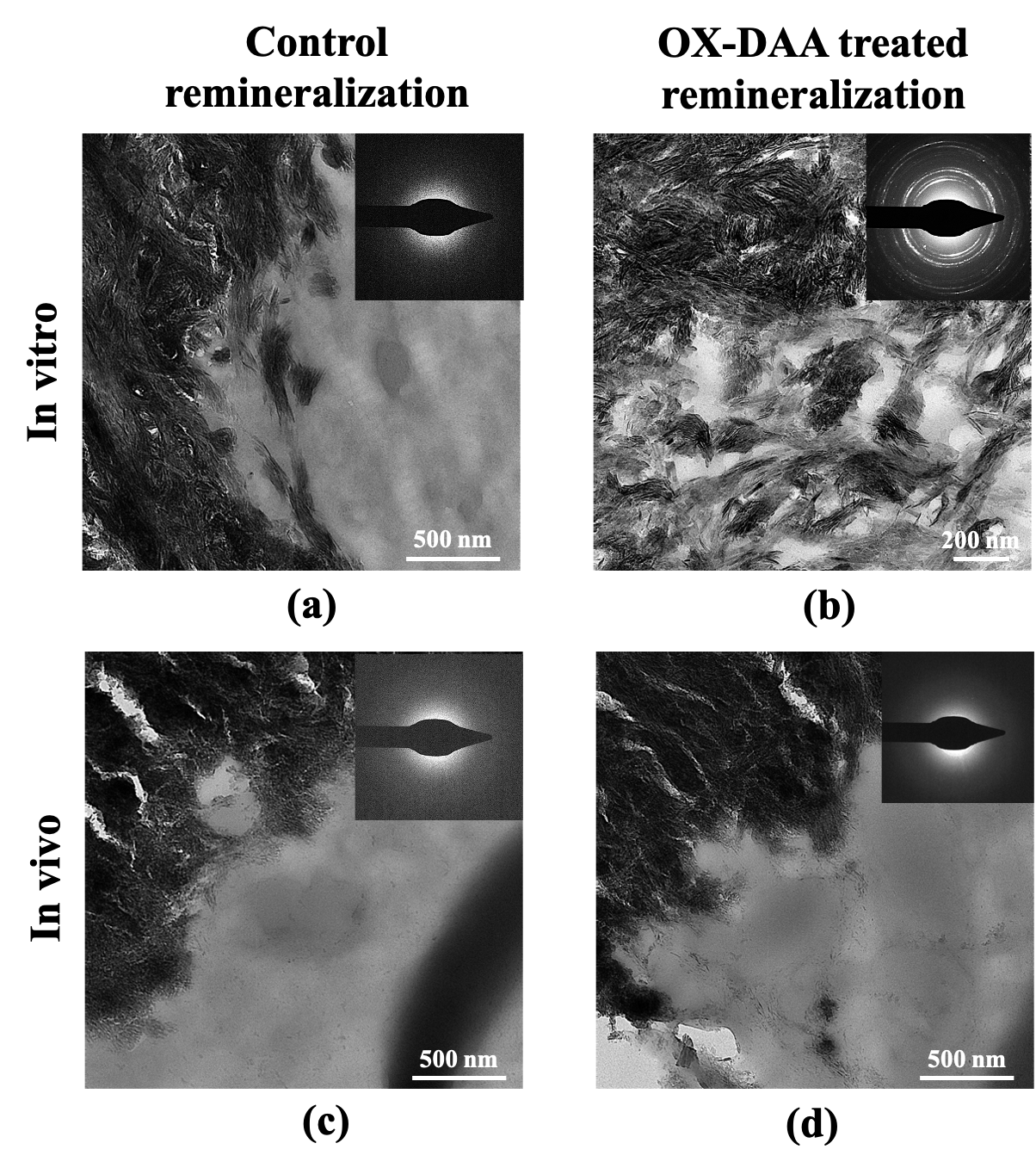


**Figure S6: In vitro and in vivo resin-dentin interface remineralization observed by TEM.** (a) After 4 weeks, TEM revealed a hybrid layer of completely demineralized dentin that was devoid of intrafibrillar and interfibrillar mineral crystallites, ﻿as demonstrated by selected-area electron diffraction (SAED). (b) After 4 weeks, the hybrid layers were almost completely remineralized in OX-DAA treated group. The intrafibrillar remineralization was observed, with an orderly arrangement of nanoplatelets that revealed the pleaded, rope-like subfibrillar architecture of the collagen fibrils, ﻿as demonstrated by the 002 Debye arc obtained from SAED of the intrafibrillar minerals. (c) After 12 days, TEM revealed a hybrid layer of completely demineralized dentin that was devoid of intrafibrillar and interfibrillar mineral crystallites in vivo, ﻿as demonstrated by selected-area electron diffraction (SAED). (d) After 12 days, the characteristic electron-dense mineral phase could be seen within the hybrid layer in OX-DAA treated group. These electron-dense mineral phases appear as chains or clusters, which appear to be aligned and oriented along demineralized collagen fibers, but SAED showed devoid of intrafibrillar and interfibrillar mineral crystallites.


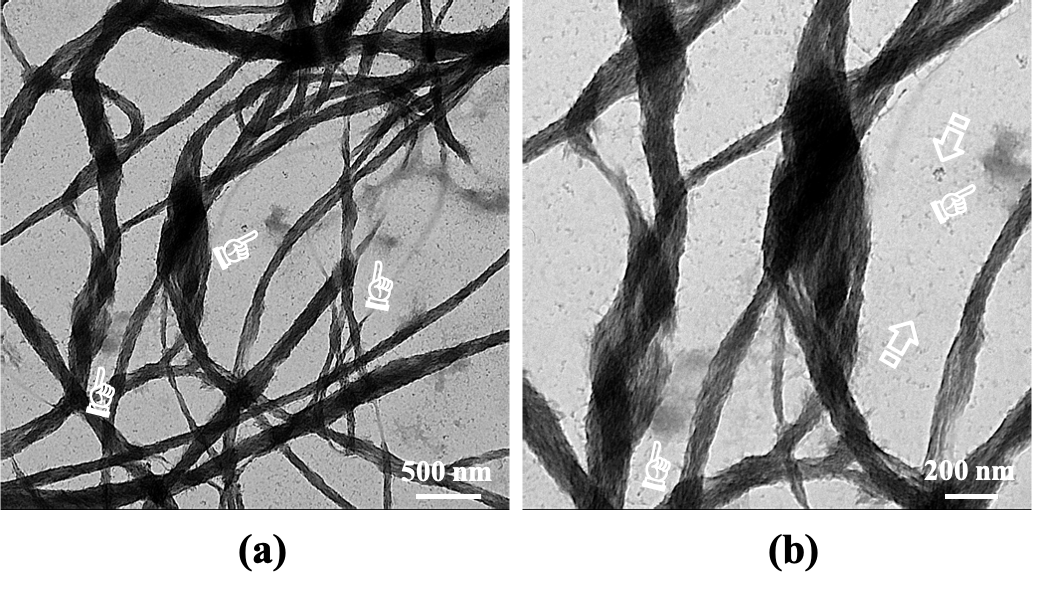


**Figure S7: TEM ﻿characterization of OX-Collagen** **collagen ﻿mineralization for 1 day.** (b) High-magnification image of (a). OX-DAA molecules existed on the surface of OX-DAA-Collagen, and the quinones in the molecules had the ability to attract calcium ions, so we could see the pool of mineralized precursor formed by the aggregation of PAA-ACP near Collagen. Mineralized precursor pool, pointer; PAA-ACP nanoparticles, open arrow.


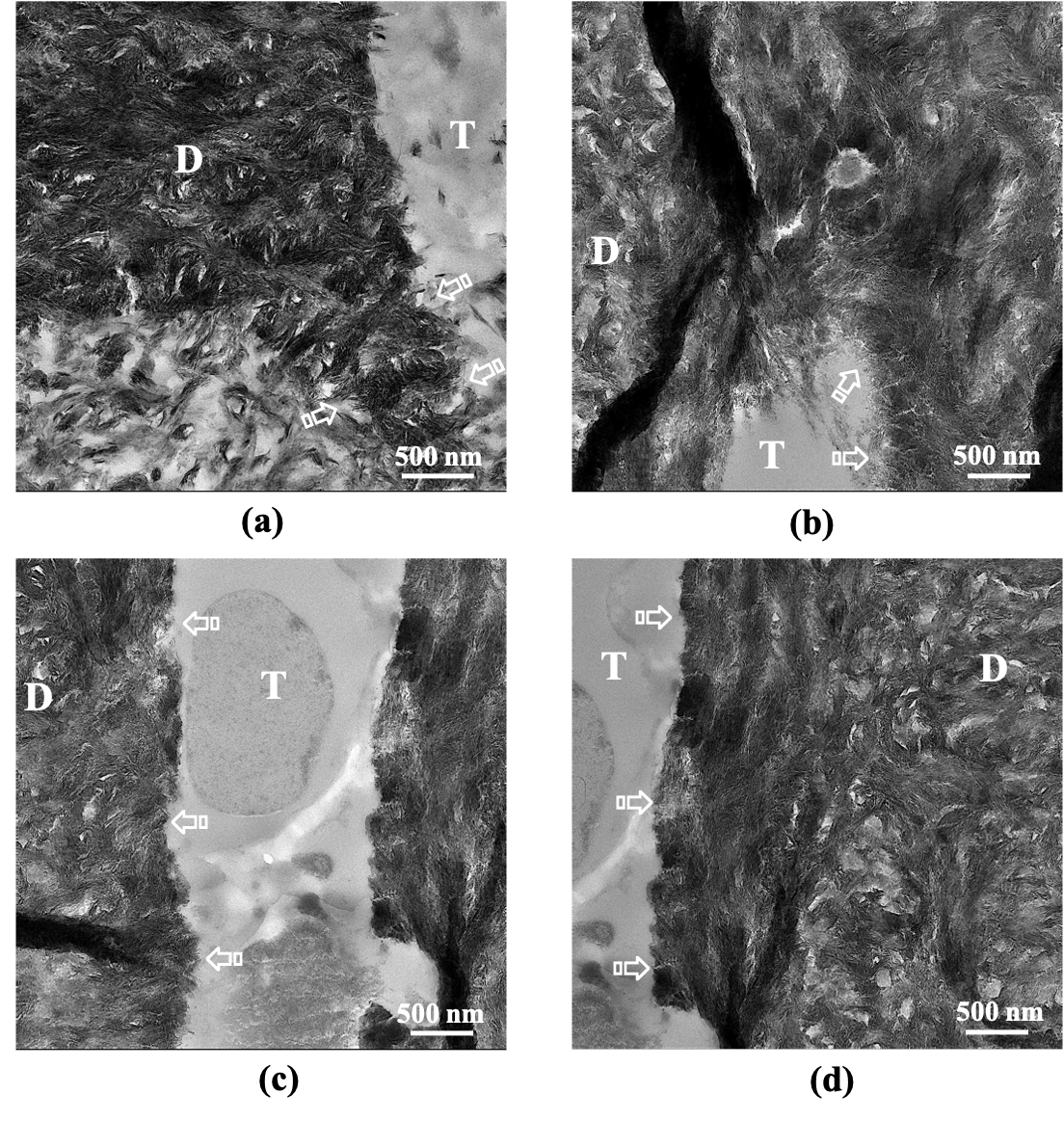


**Figure S8: In vitro resin-dentin interface remineralization observed by TEM.** D, ﻿mineralized intertubular dentin. T, dentinal tubule. (a) After 4 weeks, the hybrid layers were almost completely remineralization in OX-DAA treated group. The intrafibrillar remineralization was observed, and the demineralized dentin around the dentin tubules was more highly mineralized (open arrow). (b-d) The collagen fibers around the dentin tubules were severely mineralized (open arrow), and even new minerals were attached to the surface of the dentin tubules (d).

**References**

[1] L.P. Wu, H. Shao, Z.H. Fang, Y.C. Zhao, C.Y. Cao, Q.L. Li, “Mechanism and effects of polyphenol derivatives for modifying collagen,” ACS Biomater. Sci. Eng., vol. 5, no. 9, pp. 4272-4284, 2019. <http://dx.doi.org/10.1021/acsbiomaterials.9b00593>

[2] L.P. Wu, Q.Q. Wang, Y.Z. Li, M.M. Yang, M.L. Dong, X.X. He, S.L. Zheng, C.Y. Cao, Z. Zhou, Y.C. Zhao, Q.L. Li, “A dopamine acrylamide molecule for promoting collagen biomimetic mineralization and regulating crystal growth direction,” ACS Appl. Mater. & Inter., vol. 13, no. 33, pp. 39142-39156, 2021. <http://dx.doi.org/10.1021/acsami.1c12412>

[3] W. Chen, H.M. Jin, H. Zhang, L.P. Wu, G.Q. Chen, H. Shao, S.R. Wang, X.X. He, S.L. Zheng, C.Y. Cao, Q.L. Li, “Synergistic effects of graphene quantum dots and carbodiimide in promoting resin–dentin bond durability,” Dent. Mater., vol. 37, no. 10, pp. 1498-1510, 2021. <http://dx.doi.org/10.1016/j.dental.2021.07.004>

[4] Y. Liu, Y. Wang, “Effect of proanthocyanidins and photo-initiators on photo-polymerization of a dental adhesive,” J. Dent., vol 41, no 1, pp 71-79, 2013. <http://dx.doi.org/10.1016/j.jdent.2012.10.006>

[5] K. Li, C. Yao, Y. Sun, K. Wang, X. Wang, Z. Wang, J.K.H. Tsoi, C. Huang, C.K.Y. Yiu, “Enhancing resin-dentin bond durability using a novel mussel-inspired monomer,” Mater. Today Bio, vol. 37, pp. 100174, 2021. http://dx.doi.org/10.1016/j.mtbio.2021.100174

[6] T. Shan, L. Huang, F.R. Tay, L. Gu, “Retention of Intrafibrillar Minerals Improves Resin-Dentin Bond Durability,” J. Dent. Res., vol. 101, no. 12, pp. 1490-1498, 2022. <http://dx.doi.org/10.1177/00220345221103137>

[7] S. Armstrong, L. Breschi, M. Özcan, F. Pfefferkorn, M. Ferrari, B. Van Meerbeek, “Academy of dental materials guidance on in vitro testing of dental composite bonding effectiveness to dentin/enamel using micro-tensile bond strength (μTBS) approach,” Dent. Mater., vol. 33, no. 2, pp. 133-143, 2017. http://dx.doi.org/10.1016/j.dental.2016.11.015

[8] V.O. Pedrosa, F.M.G. França, C.P. Turssi, F.L.B.D. Amaral, L.N. Teixeira,E.F. Martinez, R.T. Basting, “Effects of caffeic acid phenethyl ester application on dentin MMP-2, stability of bond strength and failure mode of total-etch and self-etch adhesive systems,” Arch. Oral Biol., vol. 94, pp. 16-26, 2018. http://dx.doi.org/10.1016/j.archoralbio.2018.06.012

[9] C.M.P. Vidal, C. LaRoy, D. Chagas Toledo, L. da Mata Almeida, F. Qian, L.A. Hilgert, A.K. Bedran-Russo, “Hydroxy acids for adhesion to enamel and dentin: Long-term bonding performance and effect on dentin biostability,” J. Dent., vol. 107, pp. 103613, 2021. http://dx.doi.org/ 10.1016/j.jdent.2021.103613.

[10] C. C.ren-Olivé, J.M. Wieruszeski, E. Maes, C. Rolando, “Catechin and epicatechin deprotonation followed by 13C NMR,” Tetrahedron Lett., vol 43, no 25, pp. 4545-4549, 2002. http://dx.doi.org/[10.1016/S0040-4039(02)00745-1](https://doi.org/10.1016/S0040-4039(02)00745-1)
